# Osteoarthritis, labour division, and occupational specialization of the Late Shang China - insights from Yinxu (ca. 1250 - 1046 B.C.)

**DOI:** 10.1371/journal.pone.0176329

**Published:** 2017-05-02

**Authors:** Hua Zhang, Deborah C. Merrett, Zhichun Jing, Jigen Tang, Yuling He, Hongbin Yue, Zhanwei Yue, Dongya Y. Yang

**Affiliations:** 1 Department of Archaeology, Simon Fraser University, Burnaby, British Columbia, Canada; 2 SFU-JLU Joint Centre for Bioarchaeological Research, Department of Archaeology, Simon Fraser University, Burnaby, British Columbia, Canada; 3 Department of Anthropology, The University of British Columbia, Vancouver, British Columbia, Canada; 4 Institute of Archaeology, Chinese Academy of Social Sciences, Beijing, China; Seoul National University College of Medicine, REPUBLIC OF KOREA

## Abstract

This research investigates the prevalence of human osteoarthritis at Yinxu, the last capital of the Late Shang dynasty (ca. 1250–1046 B.C.), to gain insights about lifeways of early urban populations in ancient China. A total of 167 skeletal remains from two sites (Xiaomintun and Xin’anzhuang) were analyzed to examine osteoarthritis at eight appendicular joints and through three spinal osseous indicators. High osteoarthritis frequencies were found in the remains with males showing significantly higher osteoarthritis on the upper body (compared to that of the females). This distinctive pattern becomes more obvious for males from Xiaomintun. Furthermore, Xiaomintun people showed significantly higher osteoarthritis in both sexes than those from Xin’anzhuang. Higher upper body osteoarthritis is speculated to be caused by repetitive lifting and carrying heavy-weight objects, disproportionately adding more stress and thus more osseous changes to the upper than the lower body. Such lifting-carrying could be derived from intensified physical activities in general and specialized occupations in particular. Higher osteoarthritis in males may reveal a gendered division of labour, with higher osteoarthritis in Xiaomintun strongly indicating an occupational difference between the two sites. The latter speculation can be supported by the recovery of substantially more bronze-casting artifacts in Xiaomintun. It is also intriguing that relatively higher osteoarthritis was noticed in Xiaomintun females, which seems to suggest that those women might have also participated in bronze-casting activities as a “family business.” Such a family-involved occupation, if it existed, may have contributed to establishment of occupation-oriented neighborhoods as proposed by many Shang archaeologists.

## Introduction

**Fig 1:**
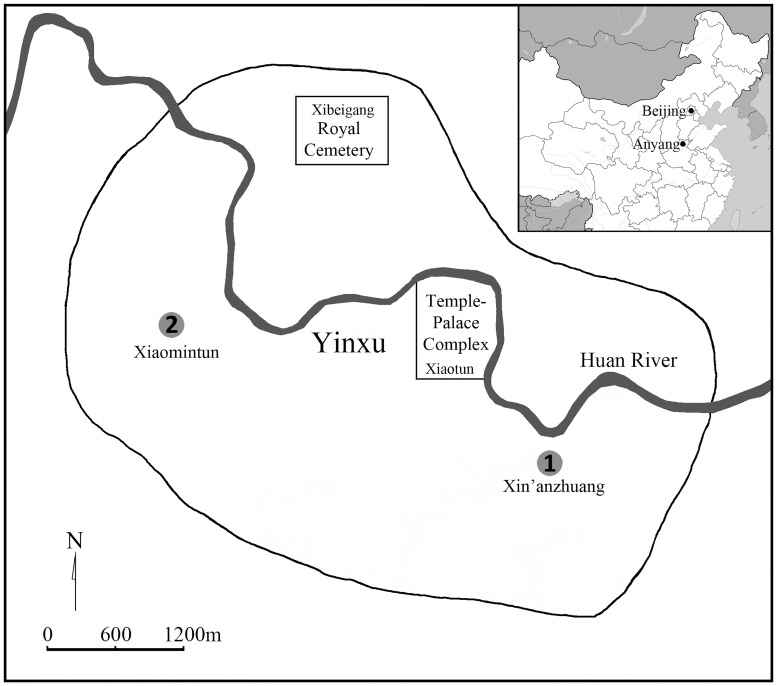
Geographic location of modern city Anyang (inset) and the two sites of this study: (1) Xin’anzhuang and (2) Xiaomintun. Adapted from [5].

The last capital city of Shang is located on both banks of the Huan River to the northwest of the modern city of Anyang, China (114.19°E, 36.07°N) (Fig 1). The archaeological area, now known as “Yinxu”, has been listed as a UNESCO World Heritage site (2006). It is well-known for the recovery of the earliest Chinese writing system, oracle bone inscriptions, as well as artifacts representing a sophisticated craft industry, especially bronze casting. Shang civilization, associated with early urbanization in China, flourished at Anyang for over 200 years (ca. 1250–1046 B.C.) [1]. This time period is generally phrased as the Late Shang in Chinese archaeology [2]. During this time, social stratification was further intensified; new occupations and urban life are believed to have emerged. Yinxu people, particularly commoners, were likely divided into different occupational groups involved in specialized craft production in order to fulfill their duties to the Shang kings and to support their families [3,4].

It is important to reconstruct how these dramatic social and cultural changes affected health status and lifeways of the Yinxu people. Such insights can prove helpful for understanding subsequent changes in the Shang Dynasty. One direct approach to the reconstruction of past human experience is the study of ancient human skeletal remains. Human bones, as a living tissue, can be shaped and crafted through adaptive responses to cultural and environmental changes [6], following the principles of bone functional adaptation [7]. One of the commonly documented and well-studied data sets in the field of human bioarchaeology is osteoarthritis. Previous clinical and osteoarchaeological studies demonstrate that osteoarthritis can be caused by many factors such as age, sex, genetics, hormones, nutrition, systemic inflammation, and body mass among others [8–12]. It is well-established that intensive physical demands and mechanical strains can prompt the development of osteoarthritis [13–17]. Some studies have also shown that an etiological relationship can be determined between certain osteoarthritis patterns and specific occupational or habitual practices [18–21]. As a result, many efforts have been made to use osteoarthritis to investigate subsistence and lifeways [20,22–26] and to reconstruct past societal changes such as gendered division of labour and occupational or habitual activity patterns [27–33].

This study will take a similar approach by examining osteoarthritis in human skeletal remains from Yinxu to explore connections between osteoarthritis frequencies and social factors such as gendered division of labour and occupational specialization. This osteoarchaeological study is also expected to shed light on the growing understanding of social dynamics in Late Shang society and the process of early urbanization in ancient China.

### Archaeological context at Yinxu

Yinxu, the Shang core area, was called the Great Settlement Shang (*dayishang*, 大邑商) in oracle bone inscriptions [34], which can be literally translated as a Big Urban Centre. Bordered by mountain ranges on the west, this urban settlement was situated on the Yellow River’s alluvial plain in North China. Fertile soil on the broad floodplain, vast forests, and diverse wildlife resources could have provided Yinxu inhabitants with significant economic advantages [35–37]. However, when Yinxu was established, the Holocene Climate Optimum was drawing to an end, with the climate gradually turning the region cooler and dryer [35,38,39]. The deteriorating climate must have exerted dramatic pressures on the urban population. Shang kings had to devote major resources to developing agriculture: some oracle bone inscriptions depict “the masses” (*ren*人 people or *zhong* 众 a group of people) working in agricultural fields [40]; archaeological evidence also indicates maximal exploitation of forest and other natural resources in the region. In addition to the drastically increased number of lithic tools for agriculture (such as knives and sickles) [41], the diversity of cultivated crop species, such as millet, rice, sorghum, barley and wheat, also increased [42–45], possibly as a risk reduction mechanism in the event of total crop failure. Recent pollen studies reveal new evidence of landscape alteration on the wetlands to the east of Yinxu [37]. This may suggest that in order to secure sufficient food resources intensive crop cultivation may have expanded beyond the existing farmland. During the rise of the urban centre, Yinxu must have witnessed an intensified labour division. Artisans were able to be free from subsistence activities in order to sustain the high levels of handicraft industries needed for manufacturing pottery, bone, jade, lithic and bronze items. Numerous artifacts were excavated from Yinxu, revealing the magnitude and scale of production that would have created different specialized occupations. A recent recovery of vast quantities of raw materials for manufacturing bone artifacts serves to emphasize the extent of output of the craft industries of the time [46,47].

The Yinxu society is considered to be organized and stratified by lineage-based groups (*zu*族) [1,4,48–51]. The kingly lineage (*wangzu*王族), princely lineages (*zizu*子族) and all other royal descendant lineages (*duozizu*多子族) were at the top of the kinship community pyramid [4,52]. Social elites, the heads of these lineages, also formed hierarchies tied to the ruling king through the closeness of kin relationships and were assigned social responsibilities to the ruling kings [49]. The non-elite members of the different lineages were “the masses” [53]. Controlled by the elites, “the masses” laboured in subsistence practice, craft production, construction, and warfare to fulfill the political and economic demands of the Shang kings and to support the rapidly expanded urban population [4,51]. This lineage system functioned not only in the social and cultural aspects of the society, but also in determining the spatial distribution of neighbourhoods [34]. These segregated neighbourhoods are thought to be associated with certain specialized occupations that can be estimated by archaeological evidence of workshops found nearby [4,51,54,55].

Previous studies of archaeological evidence and oracle bone inscriptions have suggested that such stratified social and cultural lineage-based groups affect the distribution and clusters of cemeteries at Yinxu. Recovered burial goods can often reveal the social identity of the deceased in cemeteries. Multiple lines of archaeological evidence corroborate and help determine the labour division and lifeways of recovered human skeletal individuals.

## Materials and methods

### Materials

The 167 adult skeletons included in this study were recovered from two recently excavated archaeological sites in Anyang (Fig 1): Xin’anzhuang and Xiaomintun. Since the comprehensive archaeological site reports are still in preparation for publication, we here briefly introduce the background of the sites.

1. Xin’anzhuang site, located on the south of the Palace-Temple complex of Yinxu, was excavated in 1993 and 2007. Over 300 burials of the Yinxu culture were recovered, but only 112 human skeletons were preserved well enough to be included in this study. Although this site is adjacent to several identified archaeological features such as workshops of pottery and bone artifacts, and a bronze foundry [47,55–57], no clear patterns of burial assemblages have been found that can provide strong evidence to link this cemetery population to any specialized occupation. We therefore assume them to be non-specialist commoners.
2. Xiaomintun site was excavated in 2003 and 2004 [58], revealing rich archaeological features of bronze foundries, residences and burials. Bronze-casting tools and a special pattern of burial goods found in many burials suggest their occupations were related to the nearby bronze foundry [54]. From Xiaomintun, 55 well-preserved individuals were included in this analysis.

All human remains included in this study can be securely assigned to Yinxu culture (ca. 1250 B.C.– 1046 B.C.) by characteristic features of stratigraphy, burial structures, and mortuary goods [58,59]. Compared with the large ramped richly-furnished tombs in the royal cemetery at the *Xibeigang* (西北岗) and the Palace-Temple complex at *Xiaotun* (小屯) (Fig 1), these small-sized rectangular pits are believed to be the Yinxu commoners’ burials [53] as over 95% of these burials are less than 3 m^2^ in size and most lack luxury burial goods.

### Methods

#### Ethics statement

This osteoarchaeological research of the excavated human remains was part of two collaborative studies: “The Regional Archaeological Survey in the Huan River Valley”, and “Human and Social Dynamics of Early Bronze Age China” conducted by Dr. Zhichun Jing (Department of Anthropology at the University of British Columbia) and Dr. Jigen Tang (Institute of Archaeology at the Chinese Academy of Social Sciences). The studies are officially approved by the China Bureau of Cultural Heritage Administration. The human skeletons analyzed are all archaeological samples that were excavated under supervision of Drs. Tang and Jing. All the samples are currently curated at the Anyang Work Station, a field facility of the Institute of Archaeology at the Chinese Academy of Social Sciences. Examination of the skeletal human samples was conducted at the Anyang Work Station, strictly following the standards and protocols in accordance with the WMA Declaration of Helsinki–Ethical Principles for Medical Research Involving Human Subjects.

#### Age estimation and sex identification

Multiple morphological indicators were used to estimate sex and age-at-death of each individual following the standard protocols [60]. Sex assessment was based primarily on pelvic [60–62] and cranial morphology [60]. Three categories: male, female and indeterminate were created in this study. To increase the accuracy of any sex comparisons only males and females were included, with indeterminates excluded.

Age-at-death estimation was based on features including pubic symphysis [63] and auricular surface [64], cranial suture closure [65], and dental wear pattern [66]. Since osteoarthritis is age-progressive and strongly affected by physical activities throughout adulthood, subadults (younger than 20 years) were excluded from this study. Adult individuals were classed into three age categories: young adults (20–34 years), middle adults (35–49 years), and older adults (50+ years) (Table 1). In addition, adults with indeterminate age-estimates (20+ years) were excluded from statistical analyses. Moreover, the age categories of middle (35–49 years) and older adults (50+ years) have been combined into a broader category: older adults (≥35 years) for further statistical analysis to increase sample size (Table 1). Although these two samples are not normally distributed (Shapiro-Wilkes tests: *P < 0*.*01*), the age and sex distribution of Xin’anzhuang sample does not differ significantly from the Xiaomintun sample by the Mann-Whitney test (*P > 0*.*05*).

**Table 1:** Demographic distribution of the samples analysed in this study* (see S1 Table for detailed specimen numbers).

	Xin’anzhuang (non-specialists)				Xiaomintun (bronze artisans)				Total sample			
Age Group	M	F	I	Total	M	F	I	Total	M	F	I	Total
Young adults (20–34)	20	26	5	51	14	8	1	23	34	34	6	74
Middle adults (35–49)	21	23	6	50	10	6	2	18	31	29	8	68
Old adults (50+)	1	2	1	4	3	5	0	8	4	7	1	12
Adults (20+)	1	2	4	7	1	1	4	6	2	3	8	13
Total adults	43	53	16	112	28	20	7	55	71	73	23	167

* M = Male; F = Female; I = Indeterminate sex; and Total = Total individuals including adults of indeterminate age and sex.

#### Examination criteria for osteoarthritis

Osteoarthritis was examined macroscopically with the help of a X10 magnifying hand lens. Synovial joints of the shoulder, elbow, wrist, hand, hip, knee, ankle, foot, and vertebral apophyseal facet joints were graded and recorded. Specific locations of joints for detailed observation for osteoarthritis were chosen following the description of Larsen and Kelly [67] (S2 Table). The presence of osteoarthritis was counted only when the presence of eburnation or at least two of the following criteria were observed: marginal lipping, subchondral bone porosity, new bone formation on the joint surface, and joint contour alteration [13,68]. The severity was graded as follows: 1) slight (one minor change is observed; this was not considered as presence of osteoarthritis in this study); 2) moderate (two or more criteria are exhibited); 3) severe (eburnation and any of the previous criteria) [13,68]. Osteophytosis was scored only when the marginal lipping developed horizontally to the vertebral body, in order to differentiate from the vertical ossification of anterior longitudinal ligament. The presence of Schmorl’s nodes were also assessed following the scoring criteria described in the Standards [60]: barely discernible, moderate and marked expression. Only the latter two were recorded as present. Any skeletal individuals with at least half of the articular surface of a joint element or vertebral body available for examination were included in the analysis. As a result, the total number of individuals included in this study varies slightly from one joint system to another. Any possible secondary arthritis that is most likely associated with an observed fracture on the skeleton was excluded. Due to incompleteness of the skeletons, a left and right side comparison was not conducted in this study. In order to eliminate the potential intra-observer error, the same criteria were followed by the first author (H. Zhang) to re-assess 30 randomly selected individuals from the Xin’anzhuang site at different times. Moreover, 30 randomly selected individuals were scored by the second author (D.C. Merrett) to evaluate the inter-observer error. The estimated bias caused by two observers was small and statistically insignificant (Kappa test: K = 0.755, Std. = 0.115, Approx. T = 6.057, Approx. Sig. = 0.000).

#### Statistical analysis

Multivariate odds ratios (ORs) were used to assess statistical significance for each comparison (for example, males vs. females, or Xin’anzhuang vs. Xiaomintun). Following the analytical procedures described by Klaus [69], ORs were calculated separately for each joint system in each age cohort: OR_20-34_, young adults (20–34 years); and OR_≥35_, older adults (≥35 years). A Mantel-Haenszel common odds ratio (OR_MH_) is then estimated to determine the overall prevalence pattern between two groups of people as an age-related proportion [20,69,70]. When the prevalence is higher in the first group compared (in this case, the males), OR is greater than 1; if prevalence is higher in the second group compared (the females), OR is less than 1. For example, an OR of 3.620 would mean the prevalence of osteoarthritis in this joint system is 3.620 times greater in males; an OR of 0.389 would represent the prevalence is 2.570 times (1.000/0.389 = 2.570) greater in females. OR statistic is considered to be a very useful method to help determine if the crude prevalence is indeed different between two groups, as it makes allowance for any skewness brought by non-identical age structure in archaeological skeletal samples [70]. Significant difference (*P < 0*.*05*) was determined by Pearson chi-square tests (χ^2^) or by Fisher’s exact tests when the cell number is less than 5. All analyses were conducted using SPSS 22.

## Results

### Overall crude prevalence of osteoarthritis

Overall, 45.1% of the examined adults (n = 162) were affected by osteoarthritis in the appendicular joints; 28.8% (n = 104) presented with Schmorl’s nodes and 32.7% (n = 104) displayed osteophytosis on the vertebral body; 19.2% (n = 104) suffered apophyseal joint facet osteoarthritis. In general, osteoarthritis was most common in the foot joints (specifically, the metatarsal-phalangeal joints, 52.0%, n = 100) and least common for the wrist (0%, n = 54) and hand joints (2.4%, n = 41) (Table 2). For osseous changes in the spine, very few Schmorl’s nodes were observed in cervical vertebrae (2.5%, n = 81), but were more frequently seen in thoracic (27.3%, n = 88) and lumbar vertebrae (15.7%, n = 89) (Table 2). All changes on the appendicular joints and vertebral bodies occur more frequently in older individuals in both males and females (Table 2). The crude prevalence of osteoarthritis in Xin’anzhuang (S3 Table) and in Xiaomintun (S4 Table), and chi-square test results (S5 Table) for the comparisons between and within Xin’anzhuang and Xiaomintun are presented in the Supporting Information section.

**Table 2:** Crude prevalence of osteoarthritis (Affected / Observed) by sex and age.

			Male			Female			Total		
Joint Systems^†^			Young adults	Older adults	Total*	Young adults	Older adults	Total*	Young adults^#^	Older adults^‡^	Total^§^
**Upper limb**		**Shoulder**	4/21	4/22	8/44	1/22	2/22	3/46	5/46	6/46	11/95
		**Elbow**	0/21	2/18	3/40	1/23	0/24	1/48	1/45	3/45	5/93
		**Wrist**	0/13	0/7	0/21	0/15	0/15	0/31	0/29	0/23	0/54
		**Hand**	0/10	0/5	0/15	0/14	0/11	0/25	0/24	1/17	1/41
**Lower limb**		**Hip**	1/31	3/29	4/62	2/28	2/33	4/62	3/60	6/67	9/133
		**Knee**	4/25	4/20	8/46	3/22	9/28	12/51	7/49	14/53	22/106
		**Ankle**	1/16	3/19	3/44	1/24	0/25	1/50	1/50	3/45	4/99
		**Foot**	8/21	15/19	23/41	8/22	16/24	24/46	16/45	35/49	52/100
**Spine**	**Cervical**	**S**	1/13	1/18	2/31	0/20	0/23	0/43	1/35	1/45	2/81
		**Ap**	0/14	7/19	7/33	1/20	4/25	5/45	1/36	13/48	14/85
		**Ost**	0/14	9/20	9/34	0/20	5/23	5/43	0/36	14/47	14/84
	**Thoracic**	**S**	7/20	7/19	15/40	4/23	5/22	9/45	11/44	12/43	24/88
		**Ap**	0/20	3/19	3/40	0/21	3/22	3/43	0/42	6/43	6/86
		**Ost**	0/20	7/19	7/40	1/22	8/22	9/44	1/43	15/43	16/87
	**Lumbar**	**S**	3/21	2/19	6/41	4/21	4/23	8/44	7/43	6/44	14/89
		**Ap**	0/19	2/18	2/38	1/21	6/23	7/44	1/41	9/43	10/85
		**Ost**	1/21	6/19	8/41	3/21	12/23	15/44	4/43	20/44	25/89

* Total = Total individuals including adults of indeterminate age (20+);

^#^ Young adults, including adults of indeterminate sex;

^**‡**^ Older adults, including adults of indeterminate sex;

^**§**^ Total = Total individuals including adults of indeterminate age (20+) and sex;

^**†**^ S = Schmorl’s nodes; Ap = Apophyseal facets; Ost = Vertebral body marginal osteophytosis.

### Presence of eburnation

**Fig 2:**
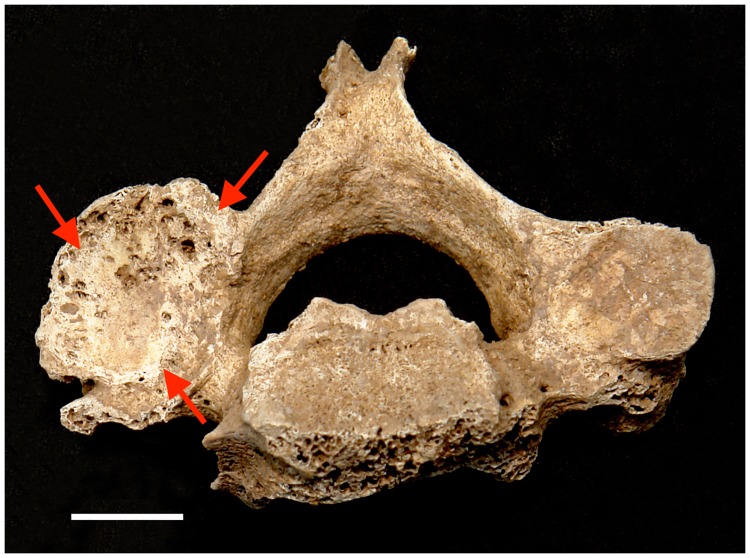
Eburnation (arrows) on the left inferior cervical apophyseal facet (2007AXAM140). Scale bar = 1cm.

**Fig 3:**
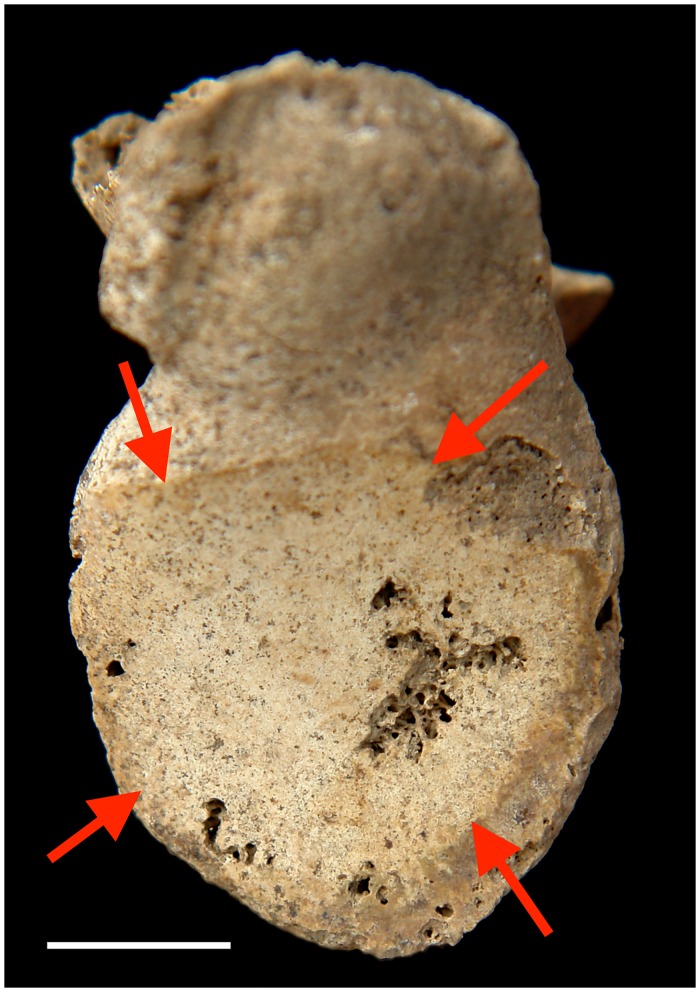
Eburnation (arrows) on the left glenoid fossa (2007AXAM165). Scale bar = 1cm.

The prevalence of eburnation (S6 Table), generally representing the most severe cases of osteoarthritis, is relatively high in cervical (8.2%, n = 85) (Fig 2) and lumbar (3.5%, n = 85) apophyseal joints, and knee (2.8%, n = 106) for the total sample. However, in general, males tend to have higher frequencies of eburnation and more joint system involvement than females. Specifically, eburnation in the shoulder (9.1%, n = 22) (Fig 3), hip (6.9%, n = 29), and ankle (5.3%, n = 19) are only observed in older males, while eburnation in lumbar apophyseal facet joints is observed only in older females (8.7%, n = 23) (S6 Table).

### Comparison of osteoarthritis by sex

**Fig 4:**
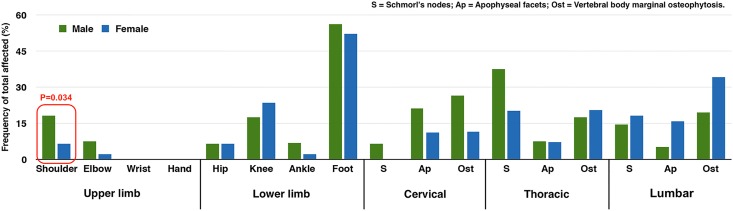
Osteoarthritis in major appendicular joints and osseous changes in the spine. Red square highlights the difference with *P* values less than 0.05. Chart is based on the age-pooled crude prevalence; *P* value is from OR analysis.

**Fig 5:**
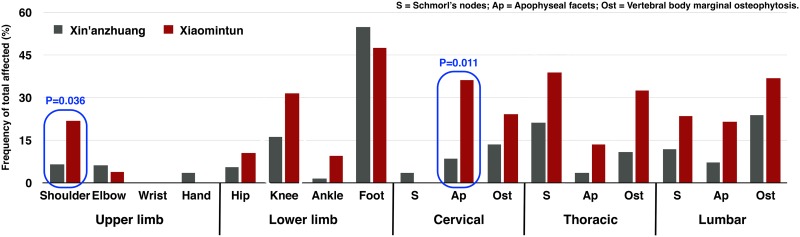
Frequency of osteoarthritis in major appendicular joints and osseous changes in the spine at Xin’anzhuang and Xiaomintun. Blue squares highlight differences with *P* values less than 0.05. Chart is based on the age-pooled crude prevalence; *P* value is from OR analysis.

**Fig 6:**
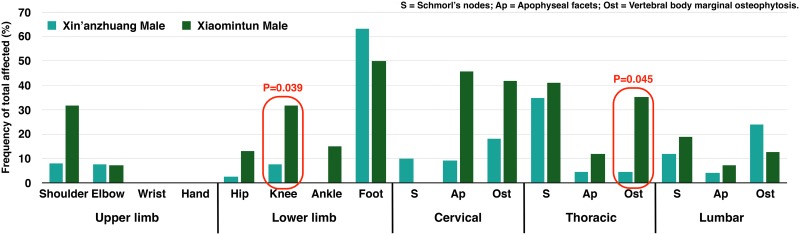
Frequency of osteoarthritis in males by site. Red squares highlight differences with *P* values less than 0.05. Chart is based on the age-pooled crude prevalence; *P* value is from OR analysis.

**Fig 7:**
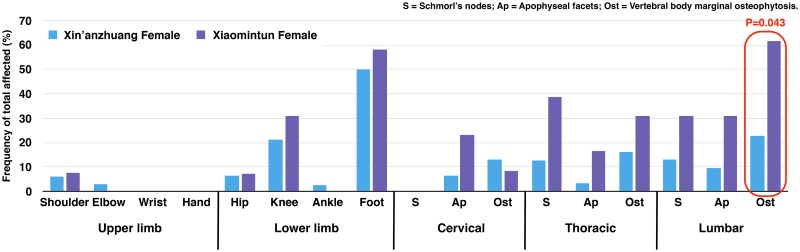
Prevalence of osteoarthritis in females by site. Red square highlights difference with *P* value less than 0.05. Chart is based on the age-pooled crude prevalence; *P* value is from OR analysis.

In order to better visually present our data and highlight the distribution of osteoarthritis, the results are presented graphically as the frequency of each joint system affected. The *P* values of significant differences obtained by OR statistics are presented in the following figures (Figs 4–7).

Overall, odds ratio statistics reveal that males present significantly greater prevalence of osteoarthritis in the shoulder (3.62 times greater than females, χ^2^ = 3.794, df = 1, *P = 0*.*034*) (Table 3) (Fig 4). Most of the nonsignificant trends toward increased osteoarthritis prevalence are also seen in males on the upper body (e.g. cervical vertebral body osteophytes: 2.95 times greater than females, χ^2^ = 1.644, df = 1, *P = 0*.*110*; thoracic apophyseal joints: 2.24 times greater than females, χ^2^ = 1.876, df = 1, *P = 0*.*107*) (Table 3). Females show non-significantly higher prevalence of osteoarthritis in the knee and lumbar spine (e.g. lumbar vertebral body osteophytes: 2.57 times greater than males, χ^2^ = 1.975, df = 1, *P = 0*.*096*) (Table 3). OR analyses also reveal that males commonly express an earlier age of onset of osteoarthritis in the shoulder, knee, and foot joints, and in Schmorl’s nodes on the thoracic vertebrae (higher prevalence of osteoarthritis in young males than in young females). The detailed odds ratio results are presented in S7 Table in the Supporting Information section.

**Table 3:** Interpretation of odds ratio results for the comparisons of osteoarthritis prevalence by sex.

Joint systems*			Total	Xin’anzhuang (non-specialists)	Xiaomintun (bronze artisans)
**Upper limb**		**Shoulder**	**3.62 times M > F**^1^	1.36 times M > F	5.17 times M > F
		**Elbow**	2.40 times M > F	1.39 times M > F	—
		**Wrist**	—	—	—
		**Hand**	—	—	—
**Lower limb**		**Hip**	1.05 times M > F	3.68 times F > M	2.70 times M > F
		**Knee**	1.36 times F > M	3.34 times F > M	1.40 times M > F
		**Ankle**	3.41 times M > F	—	—
		**Foot**	1.38 times M > F	2.33 times M > F	1.21 times F > M
**Spine**	**Cervical**	**S**	—	—	—
		**Ap**	2.22 times M > F	1.67 times M > F	2.50 times M > F
		**Ost**	2.95 times M > F^2^	1.71 times M > F	7.50 times M > F
	**Thoracic**	**S**	2.24 times M > F^3^	3.29 times M > F^4^	1.12 times M > F
		**Ap**	1.19 times M > F	1.63 times M > F	1.33 times F > M
		**Ost**	1.18 times F > M	4.20 times F > M	1.50 times M > F
	**Lumbar**	**S**	1.57 times F > M	1.45 times F > M	1.82 times F > M
		**Ap**	3.40 times F > M	2.18 times F > M	5.68 times F > M
		**Ost**	2.57 times F > M	1.05 times F > M	**15.15 times F > M**^5^

* M = Male; F = female; S = Schmorl’s nodes; Ap = Apophyseal facets; Ost = Vertebral body marginal osteophytosis;—ORs were not calculated when any cell values are zero; Bold face indicates *P* values less than 0.05.

^1^ OR_MH_ = 3.620, χ^2^ = 3.794, df = 1, *P = 0*.*034*;

^2^ OR_MH_ = 2.945, χ^2^ = 1.644, df = 1, *P = 0*.*110*;

^3^ OR_MH_ = 2.243, χ^2^ = 1.876, df = 1, *P = 0*.*107*;

^4^ OR_MH_ = 3.293, χ^2^ = 1.875, df = 1, *P = 0*.*091*;

^5^ OR_MH_ = 0.066, χ^2^ = 4.886, df = 1, *P = 0*.*015*.

For Xin’anzhuang, within-site comparison did not reveal any statistically significant difference between males and females, although Xin’anzhuang males show an elevated level of thoracic Schmorl’s nodes (3.29 times greater than females, χ^2^ = 1.875, df = 1, *P = 0*.*091*) (Table 3 and S8 Table). At Xiaomintun, males appear to exhibit a greater tendency for osteoarthritis in the shoulder, hip, knee, cervical and thoracic spine, although these differences were not significant. The only statistically significant difference was found in lumbar vertebral body rim osteophytosis prevalence at Xiaomintun, where females had a much higher frequency (15.15 times greater than males, χ^2^ = 4.886, df = 1, *P = 0*.*015*) (Table 3 and S9 Table).

### Comparison of osteoarthritis by site

Overall, odds ratio statistics reveal osteoarthritis at Xiaomintun is 4.13 times greater in the shoulder (χ^2^ = 3.454, df = 1, *P = 0*.*036*), and 5.46 times higher in cervical apophyseal joints (χ^2^ = 5.653, df = 1, *P = 0*.*011*) than in Xin’anzhuang population (Table 4 and S10 Table) (Fig 5). Moreover, the Xiaomintun sample also shows increases in knee osteoarthritis (2.67 times, χ^2^ = 2.789, df = 1, *P = 0*.*055*), in thoracic vertebral body osteophytes (3.33 times, χ^2^ = 2.521, df = 1, *P = 0*.*056*), and a higher frequency of Schmorl’s node presence in thoracic vertebrae (2.57 times, χ^2^ = 2.650, df = 1, *P = 0*.*062*) compared to the Xin’anzhuang sample (Table 4 and S10 Table).

**Table 4:** Interpretation of odds ratio results for the comparisons of osteoarthritis prevalence by site.

Joint systems*			Total	Male	Female
**Upper limb**		**Shoulder**	**4.13 times XMT > AXA**^1^	5.08 times XMT > AXA^8^	1.27 times XMT > AXA
		**Elbow**	1.31 times AXA > XMT	1.67 times XMT > AXA	—
		**Wrist**	—	—	—
		**Hand**	—	—	—
**Lower limb**		**Hip**	2.05 times XMT > AXA	6.71 times XMT > AXA	1.16 times XMT > AXA
		**Knee**	2.67 times XMT > AXA^2^	**6.29 times XMT > AXA**^9^	1.53 times XMT > AXA
		**Ankle**	6.90 times XMT > AXA^3^	—	—
		**Foot**	1.12 times AXA > XMT	2.12 times AXA > XMT	1.30 times XMT > AXA
**Spine**	**Cervical**	**S**	—	—	—
		**Ap**	**5.46 times XMT > AXA**^4^	7.52 times XMT > AXA^10^	4.07 times XMT > AXA
		**Ost**	1.50 times XMT > AXA	2.19 times XMT > AXA	2.00 times AXA > XMT
	**Thoracic**	**S**	2.57 times XMT > AXA^5^	1.49 times XMT > AXA	4.24 times XMT > AXA^12^
		**Ap**	2.93 times XMT > AXA	2.00 times XMT > AXA	4.33 times XMT > AXA
		**Ost**	3.33 times XMT > AXA^6^	**12.00 times XMT > AXA**^11^	1.90 times XMT > AXA
	**Lumbar**	**S**	2.92 times XMT > AXA^7^	2.84 times XMT > AXA	3.42 times XMT > AXA
		**Ap**	2.39 times XMT > AXA	1.00 times AXA > XMT	2.87 times XMT > AXA
		**Ost**	1.49 times XMT > AXA	2.33 times AXA > XMT	**4.78 times XMT > AXA**^13^

* AXA = Xin’anzhuang non-specialists; XMT = Xiaomintun bronze artisans; S = Schmorl’s nodes; Ap = Apophyseal facets; Ost = Vertebral body marginal osteophytosis;—ORs were not calculated when any cell values are zero; Bold face indicates p-values less than 0.05.

^1^ OR_MH_ = 0.243, χ^2^ = 3.454, df = 1, *P = 0*.*036*;

^2^ OR_MH_ = 0.374, χ^2^ = 2.789, df = 1, *P = 0*.*055*;

^3^ OR_MH_ = 0.145, χ^2^ = 1.551, df = 1, *P = 0*.*104*;

^4^ OR_MH_ = 0.183, χ^2^ = 5.653, df = 1, *P = 0*.*011*;

^5^ OR_MH_ = 0.145, χ^2^ = 1.551, df = 1, *P = 0*.*104*;

^6^ OR_MH_ = 0.300, χ^2^ = 2.521, df = 1, *P = 0*.*056*;

^7^ OR_MH_ = 0.343, χ^2^ = 1.837, df = 1, *P = 0*.*095*;

^8^ OR_MH_ = 0.197, χ^2^ = 2.301, df = 1, *P = 0*.*067*;

^9^ OR_MH_ = 0.159, χ^2^ = 3.209, df = 1, *P = 0*.*039*;

^10^ OR_MH_ = 0.133, χ^2^ = 2.119, df = 1, *P = 0*.*060*;

^11^ OR_MH_ = 0.083, χ^2^ = 2.834, df = 1, *P = 0*.*045*;

^12^ OR_MH_ = 0.236, χ^2^ = 2.155, df = 1, *P = 0*.*068*;

^13^ OR_MH_ = 0.209, χ^2^ = 2.702, df = 1, *P = 0*.*043*.

Between-site comparison reveals that Xiaomintun males exhibit significantly elevated osteoarthritis in the knee (6.29 times, χ^2^ = 3.209, df = 1, *P = 0*.*039*) and osteophytes on thoracic vertebral rims (12.00 times, χ^2^ = 2.834, df = 1, *P = 0*.*045*) (Fig 6) compared to Xin’anzhuang males. The trend is towards greater osteoarthritis frequencies in shoulder and cervical apophyseal joints (Table 4 and S11 Table). Similarly, Xiaomintun females exhibit higher frequencies of osteoarthritis in most joints than Xin’anzhuang females (Fig 7). However, only frequencies of lumbar vertebral body marginal osteophytes were increased significantly (4.78 times, χ^2^ = 2.702, df = 1, *P = 0*.*043*), while the thoracic Schmorl’s nodes were elevated to near significance (4.24 times, χ^2^ = 2.155, df = 1, *P = 0*.*068*) (Table 4 and S12 Table) in comparison to Xin’anzhuang females.

In sum, both males and females from Xiaomintun have higher osteoarthritis than their counterparts from Xin’anzhuang in most joint systems. The within-site comparisons reveal that males display even higher osteoarthritis than females at both sites, although the differences are not statistically significant.

## Discussion

### Health and stress of Yinxu commoners

Osteoarthritis has been commonly used as a general indicator of excessive physical activities in human populations in the past [71,72], although osteoarthritis presence itself may not be able to pinpoint any specific occupational stress [15,73]. The overall high prevalence of osteoarthritis across sexes and sites in this study indicates that the whole population must have undertaken continuous and heavy physical activities, which is consistent with the expected impact of early urbanization. At an urban settlement, many challenges and pressures can be associated with increased population density such as limited access to resources, and with potentially intensified social stratification. As a result, commoners are expected to work harder to survive a “stressful” urban life. Such stress has also been well illustrated in a previous study based on some of the same samples analyzed here [5]. That study presents strong evidence that childhood stress (high frequencies of linear enamel hypoplasia (LEH) and cribra orbitalia) is common among Yinxu commoners. In the present study the osteoarthritis data, reflecting habitual activities, provide not only a general picture of adult health and stress exposure, as did osteoperiostitis in the previous study [5], but also a detailed illustration of aspects of adult commoner lifeways in this urban settlement such as gendered division of labour and occupational specialization.

### Sex difference of osteoarthritis and gendered division of labour

It is well established that labour division by sex could result in the osteoarthritis differences observed between males and females [74,75]. Overall, males show higher osteoarthritis than females as males are expected to be involved in more and heavier labour activities [75]. In general, the data in this study support this assumption. However, explaining the distinctive pattern of osteoarthritis observed here is not straightforward: males present much higher osteoarthritis in the upper body joint systems of the shoulder, elbow, cervical and thoracic vertebrae than females.

It is odd, however, that the significant difference between the two sexes (sample pooled across sites) does not appear in the lower limb/body, while the opposite scenario commonly occurs clinically [76]. We believe that these observations can be effectively interpreted using a biocultural approach. Biomechanically, the human skeleton is structured to be capable of bearing more weight and stress on the lower limbs and lower vertebrae [77]. Clearly, such weight-bearing function was selected during the course of human evolution to cope with bipedalism, namely that loading increases gradually from the head to the base of the spine. In a pure biomechanical sense, we can hypothesize that osteoarthritis, resulting from cumulative degenerate changes, would emerge evenly on both the upper and lower limbs/bodies. This hypothetical even distribution of osteoarthritis would be skewed dramatically if extra and abnormal stress was added to the body, for example through culturally-specific behaviours. Human activities involving lifting/lowering, carrying, and pushing/pulling would all exert such extra loading to the human body/skeleton. Generally, these activities would add the same amount of additional compressive force to both the upper and the lower limbs and spine. However due to their structural and adaptive differences, the upper limb/body would then become more vulnerable than the lower limb/body to developing osteoarthritis in response to extra stress.

In this study, the significant difference in osteoarthritis prevalence in the upper body between males and females may suggest that, if the activities discussed above place heavy loads on the shoulder and/or head, they most likely account for the pattern observed. The sex difference of osteoarthritis in this study reveals that in gendered division of labour females were likely to be involved less in lifting and carrying, with “less” being defined as “lighter loads”, “fewer”, and/or “less frequent” activities. Rather, the slightly higher osteoarthritis prevalence of knee and lumbar spine in females may indicate that they participated in certain daily activities involving strain on the lower body. Numerous studies from other sites in China [33,78,79] and other parts of the world have demonstrated that high osteoarthritis frequency in the female lower body and/or lower spine can be caused by repetitive activities such as grain preparation [80,81]. It is reasonable to assume that labour division occurred. Indeed, certain gendered burial goods, for example the spindle whorl exclusively found in female burials at Yinxu, have been used to signify the existence of a sex-based division of labour [1].

It is clear that high osteoarthritis on the upper limb/body of Yinxu males could be caused by more arduous physical activities of men involving the use of the arms and upper body. There are many potential candidate occupations in Yinxu society, such as construction, military service, and craft activities, that could generate this impact on the skeleton. For example, excavations of Shang cultural remains, especially at Yinxu, have revealed massive earthen walls surrounding the urban centre and rammed-earth palace foundations and royal tombs [82–84]. These rammed-earth features, with stonelike hardness and durability, are built from layers of earth pounded by a bundle of wooden sticks (with a diameter of 2–5 cm each) [82,83,85]. In these labour-intensive working conditions, repetitively, with strength, and quickly lifting and throwing a wooden bundle to pound the earth could have generated tremendous pressure on the upper limb, shoulder and the neck area.

Moreover, archaeological investigations reveal that bronze weapons and tools, such as dagger axe, arrow head, spear, and knife, had been exclusively offered to the male deceased, indicating that military service and craftsmanship were probably predominant in men [1]. Oracle bone inscriptions and other historical records also document that commoners were the major on-call source of the armed forces when there was warfare [50,86]. A similar scenario has been used to interpret the observed pathological changes on bones: for example, Eng [33] suggests that the high prevalence of osteoarthritis in elbow joint in the male pastoralists in North China may result from frequently using the arms to hold weapons.

The much greater prevalence of shoulder and cervical spine osteoarthritis, and thoracic disc herniation in Yinxu males would support the abovementioned strenuous occupations. Although it is difficult to precisely pinpoint the activities of past peoples, it is possible for us to speculate the potential occupational specialization of a community if the specific archaeological context is considered.

### Occupational specialization and the nature of neighbourhoods in Yinxu society

When compared with the Xin’anzhuang sample, Xiaomintun individuals, particularly males, show an elevated prevalence of osteoarthritis on most of the appendicular joints and on the spine (Fig 5). Shoulder and cervical apophyseal facet osteoarthritis is found to be significantly different between the two samples. More detailed comparison reveals that the male cohorts contributed the difference (Fig 6). As discussed above, males tend to develop more osteoarthritis on the upper limb/body than females, reflecting labour division by sex. Therefore, these differences between the two sites may also suggest that the two skeletal populations may have undertaken biomechanically different activities associated with labour division.

An earlier age of onset of osteoarthritis is found in many joint systems in Xiaomintun young adults (e.g. shoulder, knee, foot, cervical apophyseal facets, and thoracic Schmorl’s nodes) compared to those in Xin’anzhuang (S10–S12 Tables). Clinically shoulder joints, for example, seldom seem to be affected in modern young individuals [87]. However, shoulder osteoarthritis has more often been reported among young elite athletes (particularly professional tennis players), suggesting that the repetitive strains introduced by certain exercises at a young age can cause micro-trauma in the cartilage [17]. Previous bioarchaeological studies also suggest that the high prevalence of shoulder osteoarthritis in male hunter-gatherers and agriculturalists is associated with excessive use of the joint during their daily activities [26,74,75,88]. Xiaomintun people would have to start the occupation at a very young age, so that the pathological manifestations of osteoarthritis (even eburnation) would occur by early adulthood.

**Fig 8:**
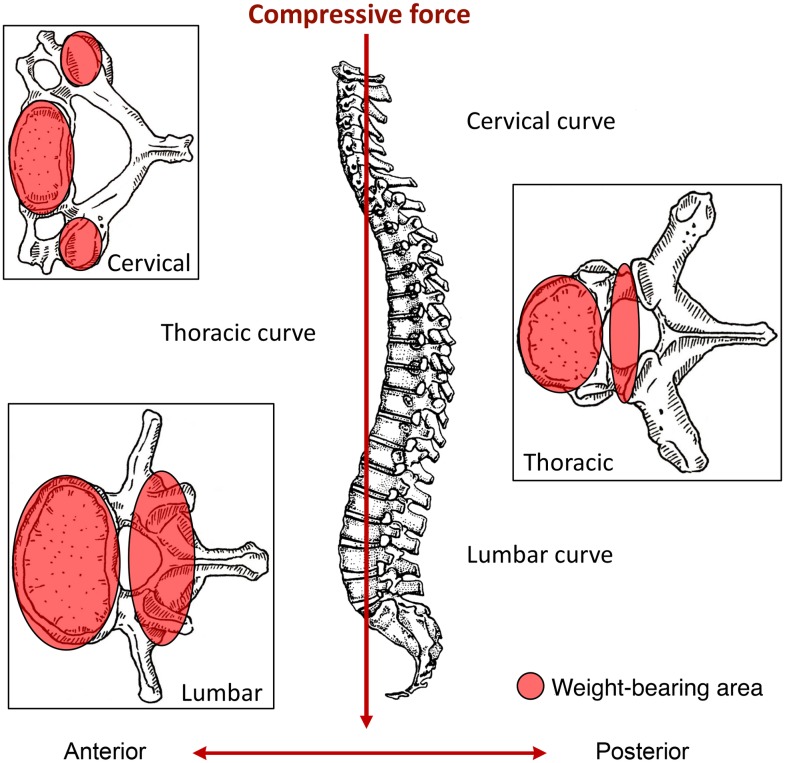
Schematic showing of direction and position of axial loading of spine (lateral view) and the location of compression force transfer on each vertebra (shadowed in red) (superior views). Due to the cervical curvature and gracile structure of the vertebral arch, cervical apophyseal joints are more vulnerable to compressive forces than the other vertebrae, whereas for thoracic, extra pressure is directed mostly toward to the vertebral body, transmitting force through the bodies, intervertebral discs, and laminae. In the lumbar region, the neural arch (pedicle and the apophyseal joints) again transmits a considerable amount of compressive force from the vertebral column to the pelvis.

Focusing on the spine, the high cervical apophyseal osteoarthritis can also be effectively interpreted from a biomechanical perspective relating the spinal curvature, the result of human bipedalism, with the specific location of stress and compression on the vertebrae (Fig 8). Because of the anterior projection in the cervical region, extra compressive force is concentrated posteriorly towards the neural arch but is transmitted anteriorly through three columns: cervical vertebral bodies and the two apophyseal facets (Fig 8) [89]. As a result, the apophyseal facets have to withstand disproportionally more stress, resulting in more frequent apophyseal osteoarthritis on cervical vertebrae [90]. This has been observed in some living populations, such as Bangladeshi professional coolies [91], Congolese wood bearers [92], and Indian foundry workers [93] who carry or lift heavy weights on the head and/or shoulder during work. In addition, the muscles of the shoulder and upper limbs would also cause extra stress on the cervical apophyseal facets, since physical activities usually generate a pulling force on the muscles and ligaments that attach to the upper spinal region (e.g. the muscles: trapezius, sternocleidomastoid, rhomboid major and minor, and levator scapulae). Moreover, bipedalism predisposes humans to suffer intervertebral disc herniation more often than non-human primates and other species [94]. However, with extra axial load-bearing, the stress certainly increases on the thoracic vertebral bodies [95,96]. Several clinic studies on military populations, who have experienced demanding physical stress involving continuous movement and flexion of the spine, have shown higher frequencies of Schmorl’s nodes [97,98]. These arguments can in fact help explain elevated levels of thoracic Schmorl’s nodes in males and the osteophytosis in the Xiaomintun people.

The detailed analysis of this osteoarthritis pattern is consistent with conclusions from the analysis above on sex-based distribution of osteoarthritis; namely, that the Yinxu commoners must have undertaken labour-demanding activities involving carrying, pushing and lifting. The observed sex and site differences suggest the existence of labour division by sex and by group. Unlike the sex dichotomy, the “group” is hard to define but it can be explored in this study through archaeological context and may be expressed by the term ‘neighbourhood’.

At Xiaomintun, the mortuary assemblages contain bronze foundry tools, special pattern of burial goods, and bronze casting-related family emblems, strongly suggesting that the occupants were likely craftsmen and artisans of the nearby foundry [58]. In contrast, Xin’anzhuang sample was assumed to be a “non-specialist” commoner group due to the lack of evidence pointing to any specific occupation. It then makes sense that the Xiaomintun bronze-casters have higher osteoarthritis in the upper body than the Xin’anzhuang non-specialists. This pattern becomes clearer when only males are compared between the two groups (Fig 6). The Xiaomintun people cannot avoid carrying and lifting heavy loads in strenuous working conditions in the bronze foundry. However, although Xin’anzhuang is situated in one of the major handicraft workshop areas at Yinxu [57] and surrounded by many kinds of manufacturing, such as bone tool and pottery workshops, not all are as physically demanding as bronze casting. Those individuals from Xin’anzhuang site could have been involved in diverse work activities, thus reducing the rapid and repetitive physical stress for developing osteoarthritis.

Although all data suggest that the occupation is male-specific, relatively higher female osteoarthritis in bronze-casting Xiaomintun group seems to indicate that the females may have also been involved in this activity-demanding heavy labour (Fig 7). We may never be able to determine the scale of participation, but we can assume that this pattern of osteoarthritis may reflect family involvement. This argument can be significant as it would allow us to speculate the existence of an expanded family/clan/lineage system in Yinxu society. Meanwhile, many Shang archaeologists argue that Yinxu society was spatially organized by lineage-based neighbourhoods [34,51], which were physically segregated but socially and economically connected [51]. Yinxu neighbourhoods have been proposed to be most likely defined by occupational specialization [3,4,51]. The osteoarthritis data in this study seem to indicate Shang cemeteries may indeed represent lineage-based and occupation-oriented neighbourhoods. Future bioarchaeological research with ancient DNA analysis and musculoskeletal entheseal changes will help to verify this argument.

It should be pointed out that the site differences by sex illustrated in Figs 5–7 are not completely consistent. We believe that this was caused by sample size and by *P* values centered on *P* ≤0.05. However, some patterns are found to be persistent throughout all three analyses: the Xiaomintun people have much higher frequencies of osteoarthritis than the Xin’anzhuang people.

One exception is that foot osteoarthritis is found at highest frequency, but no clear sex and site differences can be seen (Figs 5–7). Such a high prevalence (mainly on metatarsal-phalangeal joints) cannot be easily explained by gender division and by occupation specialization. More detailed comparisons and analyses are needed to understand the pattern of foot osteoarthritis in these assemblages.

One apparent limitation of this study is the lack of comparative samples from the pre-Shang period or from the rural region of Yinxu. Osteoarthritis in archaeological human remains in China have received considerable attention. For example, many previous studies including archaeological site reports and dissertations have documented osteoarthritis. Yet, except for a few [33,78,79,99], most of the previous studies only report typical cases. They lack population-level statistical data and a synthetic approach to link the observations with archaeological contexts. The lesions have rarely been used as activity-related stress indicators to interpret different distributions between the sexes and to investigate population-specific patterns of workload and lifestyle. More population-level comparative studies in future are essential for the exploration of past human experience in ancient China.

## Conclusions

In this study, osteoarthritis has proven to be a useful stress indicator for investigating mechanical loading and lifeways of the Yinxu people who lived during the process of early urbanization and intensifying social stratification. Overall, males show more osteoarthritis in almost all joint systems, suggesting that men were involved in more load-bearing physical activities. This pattern reveals a strong labour division by sex. Significantly elevated osteoarthritis was found on the male upper body, indicating those activities were probably primarily associated with lifting/lowering, carrying, and pushing/pulling. A significant difference in osteoarthritis was also found between the two skeletal populations Xiaomintun and Xin’anzhuang, inferring some differences in occupational specialization that are corroborated by other archaeological evidence.

This study also demonstrates that through a biocultural approach, osteological changes can be effectively used to reconstruct human activities. The biological structure of the skeleton provides a solid foundation for generating normal osseous outcomes. Significant deviations from the expected patterns of bony change can be used as evidence of culturally-influenced activities. Careful examination of the cultural context would allow for better interpretation of patterns of osteoarthritis. We found that men’s upper body suffers more osteoarthritis than women’s, which contradicts the notion that under normal conditions the lower body should develop more osteoarthritis due to human bipedalism. This can be explained if activities involved more lifting/lowering, carrying, and pushing/pulling using the upper body and shoulder than would be expected under normal circumstances. As a result, these elevated physical activities would disproportionately add more stress to the upper than the lower body, resulting in higher upper body osteoarthritis. Within the Yinxu context of early urbanization and social stratification such patterns can be derived from intensified physical activities in general and labour division and occupational specialization in particular.

Our osteoarthritis data from Xiaomintun reveal not only potential bronze-casting specialization for the site occupants but also the nature of their neighborhood. The Xiaomintun women show higher osteoarthritis than those of Xin’anzhuang, suggesting that bronze-casting might involve the whole family, not just the men. This study is generally in agreement with the notion proposed by archaeologists that Shang neighborhoods may be differentiated by specialized occupations.

This study focused on the analysis of human skeletal remains to retrieve information about variation in culturally-influenced activities in the Late Shang. New insights and possibilities are therefore proposed, subject to further cross-examination from other perspectives.

## Supporting information

S1 Table Skeletal samples used in this study. (DOCX)

S2 Table Description of joint systems and articular surfaces. (Adopted from Larsen and Kelly, 1995:109).
(DOCX)

S3 Table Crude prevalence of osteoarthritis in Xin’anzhuang by sex and age. (DOCX)

S4 Table Crude prevalence of osteoarthritis in Xiaomintun by sex and age. (DOCX)

S5 Table Results of Pearson chi-square tests for comparisons in each age cohort by sex and by site. (DOCX)

S6 Table Overall crude prevalence of eburnation by sex and age. (DOCX)

S7 Table Overall odds ratio results for the comparison of osteoarthritis prevalence between males and females. (DOCX)

S8 Table Odds ratio results for the comparison of osteoarthritis prevalence within Xin’anzhuang site by sex. (DOCX)

S9 Table Odds ratio results for the comparison of osteoarthritis prevalence within Xiaomintun site by sex. (DOCX)

S10 Table Overall odds ratio results for the comparison of osteoarthritis prevalence between Xin’anzhuang and Xiaomintun. (DOCX)

S11 Table Odds ratio results for the comparison of osteoarthritis prevalence in males between Xin’anzhuang and Xiaomintun. (DOCX)

S12 Table Odds ratio results for the comparison of osteoarthritis prevalence in females between Xin’anzhuang and Xiaomintun. (DOCX)
