# Supplementary material for: Osteoarthritis, labour division, and occupational specialization of the Late Shang China - insights from Yinxu (ca. 1250 - 1046 B.C.)
Source: PLoS One. 2017 May 2;12(5):e0176329. doi: 10.1371/journal.pone.0176329 (PMC5413014; doi:10.1371/journal.pone.0176329)
Supplement: S1 Table — (DOCX) [file pone.0176329.s001.docx]

**Supporting Information**

**S1 Table. Skeletal samples used in this study.**

| **Site** | **Specimen number** |
| --- | --- |
| **Xin’anzhuang** | 1. 2007AXAM10 |
|  | 1. 2007AXAM100 |
|  | 1. 2007AXAM101 |
|  | 1. 2007AXAM108 |
|  | 1. 2007AXAM111 |
|  | 1. 2007AXAM112 |
|  | 1. 2007AXAM113 |
|  | 1. 2007AXAM119 |
|  | 1. 2007AXAM120 |
|  | 1. 2007AXAM122 |
|  | 1. 2007AXAM123 |
|  | 1. 2007AXAM131 |
|  | 1. 2007AXAM133 |
|  | 1. 2007AXAM134 |
|  | 1. 2007AXAM136 |
|  | 1. 2007AXAM137 |
|  | 1. 2007AXAM138 |
|  | 1. 2007AXAM139 |
|  | 1. 2007AXAM140 |
|  | 1. 2007AXAM143 |
|  | 1. 2007AXAM144 |
|  | 1. 2007AXAM145 |
|  | 1. 2007AXAM146 |
|  | 1. 2007AXAM147 |
|  | 1. 2007AXAM152 |
|  | 1. 2007AXAM154 |
|  | 1. 2007AXAM157 |
|  | 1. 2007AXAM164 |
|  | 1. 2007AXAM165 |
|  | 1. 2007AXAM166 |
|  | 1. 2007AXAM168 |
|  | 1. 2007AXAM170 |
|  | 1. 2007AXAM173 |
|  | 1. 2007AXAM174 |
|  | 1. 2007AXAM177 |
|  | 1. 2007AXAM178 |
|  | 1. 2007AXAM179 |
|  | 1. 2007AXAM180 |
|  | 1. 2007AXAM181 |
|  | 1. 2007AXAM184 |
|  | 1. 2007AXAM185 |
|  | 1. 2007AXAM186 |
|  | 1. 2007AXAM187 |
|  | 1. 2007AXAM188 |
|  | 1. 2007AXAM190 |
|  | 1. 2007AXAM193 |
|  | 1. 2007AXAM194 |
|  | 1. 2007AXAM197 |
|  | 1. 2007AXAM198 |
|  | 1. 2007AXAM199 |
|  | 1. 2007AXAM200 |
|  | 1. 2007AXAM202 |
|  | 1. 2007AXAM203 |
|  | 1. 2007AXAM206 |
|  | 1. 2007AXAM209 |
|  | 1. 2007AXAM21 |
|  | 1. 2007AXAM210 |
|  | 1. 2007AXAM213 |
|  | 1. 2007AXAM215 |
|  | 1. 2007AXAM216 |
|  | 1. 2007AXAM224 |
|  | 1. 2007AXAM227 |
|  | 1. 2007AXAM228 |
|  | 1. 2007AXAM229 |
|  | 1. 2007AXAM233 |
|  | 1. 2007AXAM234 |
|  | 1. 2007AXAM237 |
|  | 1. 2007AXAM238 |
|  | 1. 2007AXAM240 |
|  | 1. 2007AXAM30 |
|  | 1. 2007AXAM35 |
|  | 1. 2007AXAM36 |
|  | 1. 2007AXAM4 |
|  | 1. 2007AXAM42 |
|  | 1. 2007AXAM43 |
|  | 1. 2007AXAM46 |
|  | 1. 2007AXAM48 |
|  | 1. 2007AXAM49 |
|  | 1. 2007AXAM50 |
|  | 1. 2007AXAM53 |
|  | 1. 2007AXAM54 |
|  | 1. 2007AXAM55 |
|  | 1. 2007AXAM59 |
|  | 1. 2007AXAM6 |
|  | 1. 2007AXAM66 |
|  | 1. 2007AXAM7 |
|  | 1. 2007AXAM8 |
|  | 1. 2007AXAM9 |
|  | 1. 2007AXAM94 |
|  | 1. 2007AXAM95 |
|  | 1. 2007AXAM99 |
|  | 1. 93AXAM126 |
|  | 1. 93AXAM140 |
|  | 1. 93AXAM142 |
|  | 1. 93AXAM154 |
|  | 1. 93AXAM155 |
|  | 1. 93AXAM188 |
|  | 1. 93AXAM218 |
|  | 1. 93AXAM220 |
|  | 1. 93AXAM222 |
|  | 1. 93AXAM239 |
|  | 1. 93AXAM265 |
|  | 1. 93AXAM268 |
|  | 1. 93AXAM269 |
|  | 1. 93AXAM300 |
|  | 1. 93AXAM301 |
|  | 1. 93AXAM317 |
|  | 1. 93AXAM342 |
|  | 1. 93AXAM352 |
|  | 1. 93AXAM362 |
|  | 1. 93AXAM378 |
|  | 1. 93AXAM390 |

| **Xiaomintun** | 1. 2003AXNM114 |
| --- | --- |
|  | 1. 2003AXNM140 |
|  | 1. 2003AXNM163 |
|  | 1. 2003AXNM171 |
|  | 1. 2003AXNM184 |
|  | 1. 2003AXNM191 |
|  | 1. 2003AXNM192 |
|  | 1. 2003AXSM204 |
|  | 1. 2003AXSM219 |
|  | 1. 2003AXSM225 |
|  | 1. 2003AXSM237 |
|  | 1. 2003AXSM354 |
|  | 1. 2003AXSM358 |
|  | 1. 2003AXSM360 |
|  | 1. 2003AXSM378 |
|  | 1. 2003AXSM385 |
|  | 1. 2003AXSM386 |
|  | 1. 2003AXSM389 |
|  | 1. 2003AXSM390 |
|  | 1. 2003AXSM399 |
|  | 1. 2003AXSM556 |
|  | 1. 2003AXSM566 |
|  | 1. 2003AXSM635 |
|  | 1. 2003AXSM636 |
|  | 1. 2003AXSM644 |
|  | 1. 2003AXSM657 |
|  | 1. 2003AXSM666 |
|  | 1. 2003AXSM667 |
|  | 1. 2003AXSM689 |
|  | 1. 2003AXSM732 |
|  | 1. 2003AXSM734 |
|  | 1. 2003AXSM736 |
|  | 1. 2003AXSM740 |
|  | 1. 2003AXSM748 |
|  | 1. 2003AXSM752 |
|  | 1. 2003AXSM756 |
|  | 1. 2003AXSM758 |
|  | 1. 2003AXSM761 |
|  | 1. 2003AXSM764 |
|  | 1. 2003AXSM769 |
|  | 1. 2003AXSM778 |
|  | 1. 2003AXSM841 |
|  | 1. 2003AXSM862 |
|  | 1. 2003AXSM877 |
|  | 1. 2003AXSM907 |
|  | 1. 2003AXSM910 |
|  | 1. 2004AXSM922 |
|  | 1. 2004AXSM928 |
|  | 1. 2004AXSM938 |
|  | 1. 2004AXSM940 |
|  | 1. 2004AXSM944 |
|  | 1. 2004AXSM964 |
|  | 1. 2004AXSM979 |
|  | 1. 2004AXSM980 |
|  | 1. 2004AXSM982 |
