# Supplementary material for: Osteoarthritis, labour division, and occupational specialization of the Late Shang China - insights from Yinxu (ca. 1250 - 1046 B.C.)
Source: PLoS One. 2017 May 2;12(5):e0176329. doi: 10.1371/journal.pone.0176329 (PMC5413014; doi:10.1371/journal.pone.0176329)
Supplement: S2 Table — (Adopted from Larsen and Kelly, 1995:109). (DOCX) [file pone.0176329.s002.docx]

**S2 Table. Description of joint systems and articular surfaces** (Adopted from Larsen and Kelly, 1995:109)**.**

| **Joint Systems** | | **Articular Surfaces** |
| --- | --- | --- |
| Upper limb | Shoulder | Humeral head; glenoid fossa |
|  | Elbow | Distal humerus (capitulum, trochlea, and coronoid fossa); proximal ulna (olecranon, coronoid process and radial notch); and head of the radius |
|  | Wrist | Distal surface of the radius and ulnar notch; distal surface of the ulna; carpals and proximal metacarpals |
|  | Hand | Metacarpal heads; phalanges |
| Lower limb | Hip | Femoral head; acetabulum |
|  | Knee | Femur distal end (lateral and medial condyles); patella; tibia proximal end (lateral and medial condyles) |
|  | Ankle | Tibia distal end (talar articular surfaces); tarsals |
|  | Foot | Metatarsal heads; phalanges |
| Spine | Schmorl’s nodes (S) | Cervical, thoracic and lumbar Schmorl’s nodes |
|  | Apophyseal Facets (Ap) | Cervical, thoracic and lumbar superior and inferior articular facets |
|  | Marginal Osteophytosis (Ost) | Cervical, thoracic and lumbar vertebral body rims |
