# Supplementary material for: Osteoarthritis, labour division, and occupational specialization of the Late Shang China - insights from Yinxu (ca. 1250 - 1046 B.C.)
Source: PLoS One. 2017 May 2;12(5):e0176329. doi: 10.1371/journal.pone.0176329 (PMC5413014; doi:10.1371/journal.pone.0176329)
Supplement: S3 Table — (DOCX) [file pone.0176329.s003.docx]

**S3 Table. Crude prevalence of osteoarthritis (Affected / Observed) in Xin’anzhuang by sex and age**.

| **Xin’anzhuang (non-specialists)** | | | | | | | | | | | |
| --- | --- | --- | --- | --- | --- | --- | --- | --- | --- | --- | --- |
|  | | | **Male** | | | **Female** | | | **Total** | | |
| **Joint Systems^†^** | | | **Young adults** | **Older adults** | **Total^*^** | **Young adults** | **Older adults** | **Total^*^** | **Young adults^#^** | **Older adults^#^** | **Total^§^** |
| **Upper limb** | | **Shoulder** | 1/12 | 1/12 | 2/25 | 1/17 | 1/15 | 2/33 | 2/32 | 2/29 | 4/63 |
|  | | **Elbow** | 0/14 | 1/11 | 2/26 | 1/18 | 0/17 | 1/36 | 1/33 | 2/31 | 4/66 |
|  | | **Wrist** | 0/10 | 0/6 | 0/17 | 0/10 | 0/11 | 0/22 | 0/21 | 0/18 | 0/41 |
|  | | **Hand** | 0/7 | 0/3 | 0/10 | 0/10 | 0/8 | 0/18 | 0/17 | 1/12 | 1/29 |
| **Lower limb** | | **Hip** | 0/19 | 1/19 | 1/39 | 2/23 | 1/24 | 3/48 | 2/43 | 3/47 | 5/95 |
|  | | **Knee** | 1/15 | 1/12 | 2/27 | 3/17 | 5/20 | 8/38 | 4/34 | 7/37 | 12/74 |
|  | | **Ankle** | 0/14 | 0/10 | 0/24 | 1/20 | 0/18 | 1/39 | 1/36 | 0/29 | 1/67 |
|  | | **Foot** | 4/11 | 8/8 | 12/19 | 6/17 | 11/17 | 17/34 | 10/29 | 23/31 | 34/62 |
| **Spine** | **Cervical** | **S** | 1/10 | 1/10 | 2/20 | 0/15 | 0/16 | 0/31 | 1/27 | 1/29 | 2/57 |
|  |  | **Ap** | 0/11 | 2/11 | 2/22 | 0/15 | 2/17 | 2/32 | 0/28 | 5/31 | 5/60 |
|  |  | **Ost** | 0/11 | 4/11 | 4/22 | 0/15 | 4/16 | 4/31 | 0/28 | 8/30 | 8/59 |
|  | **Thoracic** | **S** | 4/13 | 3/9 | 8/23 | 2/18 | 2/14 | 4/32 | 6/32 | 5/24 | 12/57 |
|  |  | **Ap** | 0/13 | 1/9 | 1/23 | 0/17 | 1/14 | 1/31 | 0/31 | 2/24 | 2/56 |
|  |  | **Ost** | 0/13 | 1/9 | 1/23 | 1/17 | 4/14 | 5/31 | 1/31 | 5/24 | 6/56 |
|  | **Lumbar** | **S** | 2/14 | 0/10 | 3/25 | 3/17 | 1/14 | 4/31 | 5/32 | 1/25 | 7/59 |
|  |  | **Ap** | 0/14 | 1/9 | 1/24 | 0/17 | 3/14 | 3/31 | 0/32 | 4/24 | 4/57 |
|  |  | **Ost** | 1/14 | 4/10 | 6/25 | 2/17 | 5/14 | 7/31 | 3/32 | 10/25 | 14/59 |

^*^ Total = Total individuals including adults of indeterminate age (20+);

^#^ Young adults, including adults of indeterminate sex; Older adults, including adults of indeterminate sex;

**^§^** Total = Total individuals including adults of indeterminate age (20+) and sex;

**^†^** S = Schmorl’s nodes; Ap = Apophyseal facets; Ost = Vertebral body marginal osteophytosis.
