# Supplementary material for: Osteoarthritis, labour division, and occupational specialization of the Late Shang China - insights from Yinxu (ca. 1250 - 1046 B.C.)
Source: PLoS One. 2017 May 2;12(5):e0176329. doi: 10.1371/journal.pone.0176329 (PMC5413014; doi:10.1371/journal.pone.0176329)
Supplement: S4 Table — (DOCX) [file pone.0176329.s004.docx]

**S4 Table. Crude prevalence of osteoarthritis (Affected / Observed) in Xiaomintun by sex and age**.

| **Xiaomintun (bronze artisans)** | | | | | | | | | | | |
| --- | --- | --- | --- | --- | --- | --- | --- | --- | --- | --- | --- |
|  | | | **Male** | | | **Female** | | | **Total** | | |
| **Joint Systems^†^** | | | **Young adults** | **Older adults** | **Total^*^** | **Young adults** | **Older adults** | **Total^*^** | **Young adults^#^** | **Older adults^#^** | **Total^§^** |
| **Upper limb** | | **Shoulder** | 3/9 | 3/10 | 6/19 | 0/5 | 1/7 | 1/13 | 3/14 | 4/17 | 7/32 |
|  | | **Elbow** | 0/7 | 1/7 | 1/14 | 0/5 | 0/7 | 0/12 | 0/12 | 1/14 | 1/27 |
|  | | **Wrist** | 0/3 | 0/1 | 0/4 | 0/5 | 0/4 | 0/9 | 0/8 | 0/5 | 0/13 |
|  | | **Hand** | 0/3 | 0/2 | 0/5 | 0/4 | 0/3 | 0/7 | 0/7 | 0/5 | 0/12 |
| **Lower limb** | | **Hip** | 1/12 | 2/10 | 3/23 | 0/5 | 1/9 | 1/14 | 1/17 | 3/20 | 4/38 |
|  | | **Knee** | 3/10 | 3/8 | 6/19 | 0/5 | 4/8 | 4/13 | 3/15 | 7/16 | 10/32 |
|  | | **Ankle** | 0/10 | 3/9 | 3/20 | 0/4 | 0/7 | 0/11 | 0/14 | 3/16 | 3/32 |
|  | | **Foot** | 4/10 | 7/11 | 11/22 | 2/5 | 5/7 | 7/12 | 6/16 | 12/18 | 18/38 |
| **Spine** | **Cervical** | **S** | 0/3 | 0/8 | 0/11 | 0/5 | 0/7 | 0/12 | 0/8 | 0/16 | 0/24 |
|  |  | **Ap** | 0/3 | 5/8 | 5/11 | 1/5 | 2/8 | 3/13 | 1/8 | 8/17 | 9/25 |
|  |  | **Ost** | 0/3 | 5/9 | 5/12 | 0/5 | 1/7 | 1/12 | 0/8 | 6/17 | 6/25 |
|  | **Thoracic** | **S** | 3/7 | 4/10 | 7/17 | 2/5 | 3/8 | 5/13 | 5/12 | 7/19 | 12/31 |
|  |  | **Ap** | 0/7 | 2/10 | 2/17 | 0/4 | 2/8 | 2/12 | 0/11 | 4/19 | 4/30 |
|  |  | **Ost** | 0/7 | 6/10 | 6/17 | 0/5 | 4/8 | 4/13 | 0/12 | 10/19 | 10/31 |
|  | **Lumbar** | **S** | 1/7 | 2/9 | 3/16 | 1/4 | 3/9 | 4/13 | 2/11 | 5/19 | 7/30 |
|  |  | **Ap** | 0/5 | 1/9 | 1/14 | 1/4 | 3/9 | 4/13 | 1/9 | 5/19 | 6/28 |
|  |  | **Ost** | 0/7 | 2/9 | 2/16 | 1/4 | 7/9 | 8/13 | 1/11 | 10/19 | 11/30 |

^*^ Total = Total individuals including adults of indeterminate age (20+);

^#^ Young adults, including adults of indeterminate sex; Older adults, including adults of indeterminate sex;

**^§^** Total = Total individuals including adults of indeterminate age (20+) and sex;

**^†^** S = Schmorl’s nodes; Ap = Apophyseal facets; Ost = Vertebral body marginal osteophytosis.
