# Supplementary material for: Osteoarthritis, labour division, and occupational specialization of the Late Shang China - insights from Yinxu (ca. 1250 - 1046 B.C.)
Source: PLoS One. 2017 May 2;12(5):e0176329. doi: 10.1371/journal.pone.0176329 (PMC5413014; doi:10.1371/journal.pone.0176329)
Supplement: S5 Table — (DOCX) [file pone.0176329.s005.docx]

**S5 Table. Results of Pearson chi-square tests for comparisons in each age cohort by sex and by site.**

|  | | | **Within-site comparison: Male vs. Female** | | | | | | | | | | | | **Between-site comparison: Xin’anzhuang vs Xiaomintun** | | | | | | | | | | | |
| --- | --- | --- | --- | --- | --- | --- | --- | --- | --- | --- | --- | --- | --- | --- | --- | --- | --- | --- | --- | --- | --- | --- | --- | --- | --- | --- |
|  | | | **Total** | | | | **Xin’anzhuang** | | | | **Xiaomintun** | | | | **Total** | | | | **Male** | | | | **Female** | | | |
|  | | | **YA** | | **OA** | | **YA** | | **OA** | | **YA** | | **OA** | | **YA** | | **OA** | | **YA** | | **OA** | | **YA** | | **OA** | |
| **Joint Systems*** | | | ***P*** | **χ^2^** | ***P*** | **χ^2^** | ***P*** | **χ^2^** | ***P*** | **χ^2^** | ***P*** | **χ^2^** | ***P*** | **χ^2^** | ***P*** | **χ^2^** | ***P*** | **χ^2^** | ***P*** | **χ^2^** | ***P*** | **χ^2^** | ***P*** | **χ^2^** | ***P*** | **χ^2^** |
| **Upper limb** | | **Shoulder** | *0.185* | 2.199 | *0.091* | 2.850 | *1.000* | 0.066 | *1.000* | 0.027 | *0.258* | 2.121 | *0.603* | 0.565 | *0.157* | 2.316 | *0.174* | 2.614 | *0.272* | 2.085 | *0.293* | 1.721 | *1.000* | 0.308 | *1.000* | 0.335 |
|  | | **Elbow** | *1.000* | 0.934 | *0.178* | 2.800 | *1.000* | 0.803 | *0.393* | 1.603 | *—* | — | *1.000* | 1.077 | *1.000* | 0.372 | *1.000* | 0.007 | *—* | — | *1.000* | 0.117 | *1.000* | 0.290 | *—* | — |
|  | | **Wrist** | *—* | — | *—* | — | *—* | — | *—* | — | *—* | — | *—* | — | *—* | — | *—* | — | *—* | — | *—* | — | *—* | — | *—* | — |
|  | | **Hand** | *—* | — | *—* | — | *—* | — | *—* | — | *—* | — | *—* | — | *—* | — | *1.000* | 0.443 | *—* | — | *—* | — | *—* | — | *—* | — |
| **Lower limb** | | **Hip** | *0.599* | 0.468 | *0.658* | 0.382 | *0.492* | 1.735 | *1.000* | 0.050 | *1.000* | 0.443 | *1.000* | 0.281 | *1.000* | 0.039 | *0.353* | 1.278 | *0.387* | 1.636 | *0.267* | 1.534 | *1.000* | 0.468 | *0.477* | 0.554 |
|  | | **Knee** | *1.000* | 0.052 | *0.351* | 0.871 | *0.603* | 0.878 | *0.370* | 1.368 | *0.505* | 1.875 | *1.000* | 0.254 | *0.660* | 0.576 | *0.090* | 3.543 | *0.267* | 2.431 | *0.255* | 2.552 | *1.000* | 1.022 | *0.371* | 1.637 |
|  | | **Ankle** | *1.000* | 1.021 | *0.073* | 4.236 | *1.000* | 0.721 | *—* | — | *—* | — | *—* | — | *1.000* | 0.397 | ***0.039*** | 5.826 | *—* | — | *0.087* | 3.958 | *1.000* | 0.209 | *—* | — |
|  | | **Foot** | *0.907* | 0.014 | *0.373* | 0.795 | *1.000* | 0.003 | *0.129* | 3.715 | *1.000* | 0.000 | *1.000* | 0.117 | *0.840* | 0.041 | *0.574* | 0.361 | *1.000* | 0.029 | *0.103* | 3.685 | *1.000* | 0.037 | *1.000* | 0.101 |
| **Spine** | **Cervical** | **S** | *0.394* | 1.587 | *0.439* | 1.310 | *0.400* | 1.562 | *0.385* | 1.664 | *—* | — | *—* | — | *1.000* | 0.305 | *1.000* | 0.564 | *1.000* | 0.847 | *0.385* | 1.664 | *—* | — | *—* | — |
|  |  | **Ap** | *1.000* | 0.721 | *0.164* | 2.501 | *—* | — | *1.000* | 0.225 | *1.000* | 0.686 | *0.315* | 2.286 | *0.222* | 3.600 | ***0.039*** | 5.319 | *—* | — | *0.074* | 3.909 | *0.250* | 3.158 | *0.570* | 0.709 |
|  |  | **Ost** | *—* | — | *0.104* | 2.636 | *—* | — | *0.675* | 0.404 | *—* | — | *0.145* | 2.861 | *—* | — | *0.534* | 0.386 | *—* | — | *0.653* | 0.737 | *—* | — | *1.000* | 0.329 |
|  | **Thoracic** | **S** | *0.187* | 1.742 | *0.322* | 0.981 | *0.208* | 1.869 | *0.343* | 1.168 | *1.000* | 0.010 | *1.000* | 0.012 | *0.139* | 2.444 | *0.245* | 1.351 | *0.651* | 0.292 | *1.000* | 0.090 | *0.194* | 2.273 | *0.309* | 1.562 |
|  |  | **Ap** | *—* | — | *1.000* | 0.038 | *—* | — | *1.000* | 0.109 | *—* | — | *1.000* | 0.064 | *—* | — | *0.380* | 1.429 | *—* | — | *1.000* | 0.281 | *—* | — | *0.527* | 1.378 |
|  |  | **Ost** | *1.000* | 0.931 | *0.975* | 0.001 | *1.000* | 0.791 | *0.611* | 0.982 | *—* | — | *1.000* | 0.180 | *1.000* | 0.396 | ***0.030*** | 4.721 | *—* | — | *0.057* | 4.866 | *1.000* | 0.308 | *0.386* | 1.010 |
|  | **Lumbar** | **S** | *1.000* | 0.171 | *0.673* | 0.400 | *1.000* | 0.064 | *1.000* | 0.745 | *1.000* | 0.196 | *1.000* | 0.277 | *1.000* | 0.039 | *0.070* | 4.565 | *1.000* | 0.000 | *0.211* | 2.484 | *1.000* | 0.114 | *0.260* | 2.616 |
|  |  | **Ap** | *1.000* | 0.928 | *0.429* | 1.442 | *—* | — | *1.000* | 0.406 | *0.444* | 1.406 | *0.576* | 1.286 | *0.220* | 3.644 | *0.477* | 0.597 | *—* | — | *1.000* | 0.000 | *0.190* | 4.463 | *0.643* | 0.403 |
|  |  | **Ost** | *0.606* | 1.105 | *0.179* | 1.802 | *1.000* | 0.188 | *1.000* | 0.046 | *0.364* | 1.925 | ***0.057*** | 5.556 | *0.978* | 0.001 | *0.405* | 0.695 | *1.000* | 0.525 | *0.628* | 0.693 | *0.489* | 0.463 | *0.089* | 3.884 |

* YA = Young adults; OA = Older adults; S = Schmorl’s nodes; Ap = Apophyseal facets; Ost = Vertebral body marginal osteophytosis; — Statistical analysis was not conducted when two or more cell values are zero; Bold face highlights the p-values less than 0.05.
