# Supplementary material for: Osteoarthritis, labour division, and occupational specialization of the Late Shang China - insights from Yinxu (ca. 1250 - 1046 B.C.)
Source: PLoS One. 2017 May 2;12(5):e0176329. doi: 10.1371/journal.pone.0176329 (PMC5413014; doi:10.1371/journal.pone.0176329)
Supplement: S6 Table — (DOCX) [file pone.0176329.s006.docx]

**S6 Table. Overall crude prevalence of eburnation (Affected / Observed) by sex and age.**

| **Eburnation** | | | **Male** | | | **Female** | | | **Total** | | |
| --- | --- | --- | --- | --- | --- | --- | --- | --- | --- | --- | --- |
| **Joint systems** | |  | **YA^a^** | **OA^a^** | **Total^a^** | **YA^a^** | **OA^a^** | **Total^a^** | **YA^b^** | **OA^b^** | **Total^c^** |
| **Upper limb** | | **Shoulder** | 0/21 | 2/22 | 2/44 | 0/22 | 0/22 | 0/46 | 0/46 | 2/46 | 2/95 |
|  | | **Elbow** | 0/21 | 0/18 | 0/40 | 0/23 | 0/24 | 0/48 | 0/45 | 0/45 | 0/93 |
|  | | **Wrist** | 0/13 | 0/7 | 0/21 | 0/15 | 0/15 | 0/31 | 0/29 | 0/23 | 0/54 |
|  | | **Hand** | 0/10 | 0/5 | 0/15 | 0/14 | 0/11 | 0/25 | 0/24 | 1/17 | 1/41 |
| **Lower limb** | | **Hip** | 0/31 | 2/29 | 2/62 | 0/28 | 0/33 | 0/62 | 0/60 | 3/67 | 3/133 |
|  | | **Knee** | 1/25 | 0/20 | 1/46 | 1/22 | 1/28 | 2/51 | 2/49 | 1/53 | 3/106 |
|  | | **Ankle** | 0/24 | 1/19 | 1/44 | 0/24 | 0/25 | 0/50 | 0/50 | 1/45 | 1/99 |
|  | | **Foot** | 1/21 | 0/19 | 1/41 | 1/22 | 0/24 | 1/46 | 2/45 | 0/49 | 2/100 |
| **Spine** | **Apophyseal facets** | **Cervical** | 0/14 | 3/19 | 3/33 | 0/20 | 3/25 | 3/45 | 0/36 | 7/48 | 7/85 |
|  |  | **Thoracic** | 0/20 | 0/19 | 0/45 | 0/21 | 0/22 | 0/43 | 0/42 | 0/43 | 0/86 |
|  |  | **Lumbar** | 0/19 | 0/18 | 0/38 | 0/21 | 2/23 | 2/44 | 0/41 | 3/43 | 3/85 |

^a^ YA = Young adults; OA = Old adults; Total = Total individuals including adults of indeterminate age (20+);

^b^ YA = Young adults including adults of indeterminate sex; OA = Old adults including adults of indeterminate sex;

^c^ Total = Total individuals including adults of indeterminate age (20+) and sex.
