# Supplementary material for: Osteoarthritis, labour division, and occupational specialization of the Late Shang China - insights from Yinxu (ca. 1250 - 1046 B.C.)
Source: PLoS One. 2017 May 2;12(5):e0176329. doi: 10.1371/journal.pone.0176329 (PMC5413014; doi:10.1371/journal.pone.0176329)
Supplement: S7 Table — (DOCX) [file pone.0176329.s007.docx]

**S7 Table. Overall odds ratio results for the comparison of osteoarthritis prevalence between males and females.**

| **Joint systems*** | | | **OR_20-34_** | **OR**_≥_ **_35_** | **OR_MH_** | ***P*** | **χ^2^** | **df** | **Interpretation**  **Male (M) vs. Female (F)** |
| --- | --- | --- | --- | --- | --- | --- | --- | --- | --- |
| Upper limb | | Shoulder | **4.941** | **3.185** | **3.620** | ***0.034*** | **3.794** | **1** | **3.620 times M > F** |
|  | | Elbow | — | — | 2.395 | *0.463* | 0.038 | 1 | 2.395 times M > F |
|  | | Wrist | — | — | — | *—* | — | — | — |
|  | | Hand | — | — | — | *—* | — | — | — |
| Lower limb | | Hip | 0.433 | 1.788 | 1.046 | *0.951* | 0.091 | 1 | 1.046 times M > F |
|  | | Knee | 1.206 | 0.528 | 0.737 | *0.558* | 0.106 | 1 | 1.357 times F > M |
|  | | Ankle | — | — | 3.409 | *0.265* | 0.522 | 1 | 3.409 times M > F |
|  | | Foot | 1.077 | 1.875 | 1.381 | *0.490* | 0.205 | 1 | 1.381 times M > F |
| Spine | Cervical | S | — | — | — | *—* | — | — | — |
|  |  | Ap | — | 3.063 | 2.223 | *0.226* | 0.774 | 1 | 2.223 times M > F |
|  |  | Ost | — | 2.945 | 2.945 | *0.110* | 1.644 | 1 | 2.945 times M > F |
|  | Thoracic | S | 2.558 | 1.938 | 2.243 | *0.107* | 1.876 | 1 | 2.243 times M > F |
|  |  | Ap | — | 1.188 | 1.188 | *0.846* | 0.060 | 1 | 1.188 times M > F |
|  |  | Ost | — | 1.021 | 0.848 | *0.792* | 0.002 | 1 | 1.179 times F > M |
|  | Lumbar | S | 0.708 | 0.559 | 0.636 | *0.465* | 0.182 | 1 | 1.572 times F > M |
|  |  | Ap | — | 0.354 | 0.294 | *0.159* | 1.180 | 1 | 3.401 times F > M |
|  |  | Ost | 0.300 | 0.423 | 0.389 | *0.096* | 1.975 | 1 | 2.570 times F > M |

* OR_20-34_, the odds ratio for young adults (20-34 years); OR_≥ 35_, the odds ratio for older adults (≥ 35 years); OR_MH_, the Mantel-Haenszel common odds ratio of each joint system; — ORs were not calculated when any cell values are zero; S = Schmorl’s nodes; Ap = Apophyseal facets; Ost = Vertebral body marginal osteophytosis; Bold face indicates p-values less than 0.05.
