# Supplementary material for: Osteoarthritis, labour division, and occupational specialization of the Late Shang China - insights from Yinxu (ca. 1250 - 1046 B.C.)
Source: PLoS One. 2017 May 2;12(5):e0176329. doi: 10.1371/journal.pone.0176329 (PMC5413014; doi:10.1371/journal.pone.0176329)
Supplement: S8 Table — (DOCX) [file pone.0176329.s008.docx]

**S8 Table. Odds ratio results for the comparison of osteoarthritis prevalence within Xin’anzhuang site by sex.**

| **Xin’anzhuang Joint systems*** | | | **OR_20-34_** | **OR**_≥_ **_35_** | **OR_MH_** | ***P*** | **χ^2^** | **df** | **Interpretation**  **Male (M) vs. Female (F)** |
| --- | --- | --- | --- | --- | --- | --- | --- | --- | --- |
| **Upper limb** | | **Shoulder** | 1.455 | 1.237 | 1.360 | *0.767* | 0.050 | 1 | 1.36 times M > F |
|  | | **Elbow** | — | — | 1.388 | *0.813* | 0.225 | 1 | 1.39 times M > F |
|  | | **Wrist** | — | — | — | *—* | — | — | — |
|  | | **Hand** | — | — | — | *—* | — | — | — |
| **Lower limb** | | **Hip** | — | 0.772 | 0.272 | *0.290* | 0.323 | 1 | 3.68 times F > M |
|  | | **Knee** | 0.333 | 0.273 | 0.299 | *0.151* | 1.270 | 1 | 3.34 times F > M |
|  | | **Ankle** | — | — | — | *—* | — | — | — |
|  | | **Foot** | 1.048 | — | 2.328 | *0.213* | 0.848 | 1 | 2.33 times M > F |
| **Spine** | **Cervical** | **S** | — | — | — | *—* | — | — | — |
|  |  | **Ap** | — | 1.667 | 1.667 | *0.638* | 0.006 | 1 | 1.67 times M > F |
|  |  | **Ost** | — | 1.714 | 1.714 | *0.527* | 0.041 | 1 | 1.71 times M > F |
|  | **Thoracic** | **S** | 3.566 | 3.000 | 3.293 | *0.091* | 1.875 | 1 | 3.29 times M > F |
|  |  | **Ap** | — | 1.625 | 1.625 | *0.744* | 1.625 | 1 | 1.63 times M > F |
|  |  | **Ost** | — | 0.313 | 0.238 | *0.225* | 0.649 | 1 | 4.20 times F > M |
|  | **Lumbar** | **S** | 0.778 | — | 0.572 | *0.552* | 0.023 | 1 | 1.45 times F > M |
|  |  | **Ap** | — | 0.458 | 0.458 | *0.531* | 0.005 | 1 | 2.18 times F > M |
|  |  | **Ost** | 0.577 | 1.200 | 0.950 | *0.941* | 0.074 | 1 | 1.05 times F > M |

* OR_20-34,_ the odds ratio for young adults (20-34 years); OR_≥ 35,_ the odds ratio for older adults (≥ 35 years); OR_MH_, the Mantel-Haenszel common odds ratio of each joint system; — ORs were not calculated when any cell values are zero; S = Schmorl’s nodes; Ap = Apophyseal facets; Ost = Vertebral body marginal osteophytosis.
