# Supplementary material for: Osteoarthritis, labour division, and occupational specialization of the Late Shang China - insights from Yinxu (ca. 1250 - 1046 B.C.)
Source: PLoS One. 2017 May 2;12(5):e0176329. doi: 10.1371/journal.pone.0176329 (PMC5413014; doi:10.1371/journal.pone.0176329)
Supplement: S9 Table — (DOCX) [file pone.0176329.s009.docx]

**S9 Table. Odds ratio results for the comparison of osteoarthritis prevalence within Xiaomintun site by sex.**

| **Xiaomintun Joint systems*** | | | **OR_20-34_** | **OR**_≥_ **_35_** | **OR_MH_** | ***P*** | **χ^2^** | **df** | **Interpretation**  **Male (M) vs. Female (F)** |
| --- | --- | --- | --- | --- | --- | --- | --- | --- | --- |
| **Upper limb** | | **Shoulder** | — | 2.571 | 5.173 | *0.160* | 1.084 | 1 | 5.17 times M > F |
|  | | **Elbow** | — | — | — | *—* | — | — | — |
|  | | **Wrist** | — | — | — | *—* | — | — | — |
|  | | **Hand** | — | — | — | *—* | — | — | — |
| **Lower limb** | | **Hip** | — | 2.000 | 2.699 | *0.436* | 0.053 | 1 | 2.70 times M > F |
|  | | **Knee** | — | 0.600 | 1.400 | *0.686* | 0.000 | 1 | 1.40 times M > F |
|  | | **Ankle** | — | — | — | *—* | — | — | — |
|  | | **Foot** | 1.000 | 0.700 | 0.826 | *0.801* | 0.015 | 1 | 1.21 times F > M |
| **Spine** | **Cervical** | **S** | — | — | — | *—* | — | — | — |
|  |  | **Ap** | — | 5.000 | 2.500 | *0.312* | 0.304 | 1 | 2.50 times M > F |
|  |  | **Ost** | — | 7.500 | 7.500 | *0.113* | 1.286 | 1 | 7.50 times M > F |
|  | **Thoracic** | **S** | 1.125 | 1.111 | 1.117 | *0.884* | 0.049 | 1 | 1.12 times M > F |
|  |  | **Ap** | — | 0.750 | 0.750 | *0.800* | 0.095 | 1 | 1.33 times F > M |
|  |  | **Ost** | — | 1.500 | 1.500 | *0.672* | 0.003 | 1 | 1.50 times M > F |
|  | **Lumbar** | **S** | 0.500 | 0.571 | 0.549 | *0.498* | 0.054 | 1 | 1.82 times F > M |
|  |  | **Ap** | — | 0.250 | 0.176 | *0.153* | 1.041 | 1 | 5.68 times F > M |
|  |  | **Ost** | **—** | **0.082** | **0.066** | ***0.015*** | **4.886** | **1** | **15.15 times F > M** |

* OR_20-34,_ the odds ratio for young adults (20-34 years); OR_≥ 35,_ the odds ratio for older adults (≥ 35 years); OR_MH_, the Mantel-Haenszel common odds ratio of each joint system; — ORs were not calculated when any cell values are zero; S = Schmorl’s nodes; Ap = Apophyseal facets; Ost = Vertebral body marginal osteophytosis; Bold face indicates p-values less than 0.05.
