# Supplementary material for: Osteoarthritis, labour division, and occupational specialization of the Late Shang China - insights from Yinxu (ca. 1250 - 1046 B.C.)
Source: PLoS One. 2017 May 2;12(5):e0176329. doi: 10.1371/journal.pone.0176329 (PMC5413014; doi:10.1371/journal.pone.0176329)
Supplement: S10 Table — (DOCX) [file pone.0176329.s010.docx]

**S10 Table. Overall odds ratio results for the comparison of osteoarthritis prevalence between Xin’anzhuang and Xiaomintun.**

| **Joint systems*** | | | **OR_20-34_** | **OR**_≥_ **_35_** | **OR_MH_** | ***P*** | **χ^2^** | **df** | **Interpretation**  **Xin’anzhuang (AXA) vs. Xiaomintun (XMT)** |
| --- | --- | --- | --- | --- | --- | --- | --- | --- | --- |
| **Upper limb** | | **Shoulder** | **0.244** | **0.241** | **0.242** | ***0.036*** | **3.454** | **1** | **4.13 times XMT > AXA** |
|  | | **Elbow** | — | 0.897 | 1.310 | *0.821* | 0.111 | 1 | 1.31 times AXA > XMT |
|  | | **Wrist** | — | — | — | *—* | — | — | — |
|  | | **Hand** | — | — | — | *—* | — | — | — |
| **Lower limb** | | **Hip** | 0.780 | 0.386 | 0.488 | *0.308* | 0.422 | 1 | 2.05 times XMT > AXA |
|  | | **Knee** | 0.533 | 0.300 | 0.374 | *0.055* | 2.789 | 1 | 2.67 times XMT > AXA |
|  | | **Ankle** | — | — | 0.145 | *0.104* | 1.551 | 1 | 6.90 times XMT > AXA |
|  | | **Foot** | 0.877 | 1.438 | 1.122 | *0.802* | 0.000 | 1 | 1.12 times AXA > XMT |
| **Spine** | **Cervical** | **S** | — | — | — | *—* | — | — | — |
|  |  | **Ap** | **—** | **0.216** | **0.183** | ***0.011*** | **5.653** | **1** | **5.46 times XMT > AXA** |
|  |  | **Ost** | — | 0.667 | 0.667 | *0.535* | 0.082 | 1 | 1.50 times XMT > AXA |
|  | **Thoracic** | **S** | 0.323 | 0.451 | 0.389 | *0.062* | 2.650 | 1 | 2.57 times XMT > AXA |
|  |  | **Ap** | — | 0.341 | 0.341 | *0.246* | 0.553 | 1 | 2.93 times XMT > AXA |
|  |  | **Ost** | — | 0.237 | 0.300 | *0.056* | 2.521 | 1 | 3.33 times XMT > AXA |
|  | **Lumbar** | **S** | 0.833 | 0.117 | 0.343 | *0.095* | 1.837 | 1 | 2.92 times XMT > AXA |
|  |  | **Ap** | — | 0.560 | 0.419 | *0.217* | 0.864 | 1 | 2.39 times XMT > AXA |
|  |  | **Ost** | 1.034 | 0.600 | 0.672 | *0.463* | 0.205 | 1 | 1.49 times XMT > AXA |

* OR_20-34,_ the odds ratio for young adults (20-34 years); OR_≥ 35,_ the odds ratio for older adults (≥ 35 years); OR_MH_, the Mantel-Haenszel common odds ratio of each joint system; — ORs were not calculated when any cell values are zero; S = Schmorl’s nodes; Ap = Apophyseal facets; Ost = Vertebral body marginal osteophytosis; Bold face indicates p-values less than 0.05.
