# Supplementary material for: Osteoarthritis, labour division, and occupational specialization of the Late Shang China - insights from Yinxu (ca. 1250 - 1046 B.C.)
Source: PLoS One. 2017 May 2;12(5):e0176329. doi: 10.1371/journal.pone.0176329 (PMC5413014; doi:10.1371/journal.pone.0176329)
Supplement: S11 Table — (DOCX) [file pone.0176329.s011.docx]

**S11 Table. Odds ratio results for the comparison of osteoarthritis prevalence in males between Xin’anzhuang and Xiaomintun.**

| **Male Joint systems*** | | | **OR_20-34_** | **OR**_≥_ **_35_** | **OR_MH_** | ***P*** | **χ^2^** | **df** | **Interpretation**  **Xin’anzhuang (AXA) vs. Xiaomintun (XMT)** |
| --- | --- | --- | --- | --- | --- | --- | --- | --- | --- |
| **Upper limb** | | **Shoulder** | 0.182 | 0.212 | 0.197 | *0.067* | 2.301 | 1 | 5.08 times XMT > AXA |
|  | | **Elbow** | — | 0.600 | 0.600 | *0.734* | 0.172 | 1 | 1.67 times XMT > AXA |
|  | | **Wrist** | — | — | — | *—* | — | — | — |
|  | | **Hand** | — | — | — | *—* | — | — | — |
| **Lower limb** | | **Hip** | — | 0.222 | 0.149 | *0.122* | 1.342 | 1 | 6.71 times XMT > AXA |
|  | | **Knee** | **0.167** | **0.152** | **0.159** | ***0.039*** | **3.209** | **1** | **6.29 times XMT > AXA** |
|  | | **Ankle** | — | — | — | *—* | — | — | — |
|  | | **Foot** | 0.857 | — | 2.120 | *0.308* | 0.468 | 1 | 2.12 times AXA > XMT |
| **Spine** | **Cervical** | **S** | — | — | — | *—* | — | — | — |
|  |  | **Ap** | **—** | 0.133 | 0.133 | *0.060* | 2.119 | 1 | 7.52 times XMT > AXA |
|  |  | **Ost** | — | 0.457 | 0.457 | *0.394* | 0.157 | 1 | 2.19 times XMT > AXA |
|  | **Thoracic** | **S** | 0.593 | 0.750 | 0.669 | *0.555* | 0.059 | 1 | 1.49 times XMT > AXA |
|  |  | **Ap** | — | 0.500 | 0.500 | *0.600* | 0.009 | 1 | 2.00 times XMT > AXA |
|  |  | **Ost** | **—** | **0.083** | **0.083** | ***0.045*** | **2.834** | **1** | **12.00 times XMT > AXA** |
|  | **Lumbar** | **S** | 1.000 | — | 0.352 | *0.306* | 0.285 | 1 | 2.84 times XMT > AXA |
|  |  | **Ap** | — | 1.000 | 1.000 | *1.000* | 0.000 | 1 | 1.00 times AXA > XMT |
|  |  | **Ost** | — | 2.333 | 2.861 | *0.295* | 0.350 | 1 | 2.33 times AXA > XMT |

* OR_20-34,_ the odds ratio for young adults (20-34 years); OR_≥ 35,_ the odds ratio for older adults (≥ 35 years); OR_MH_, the Mantel-Haenszel common odds ratio of each joint system; — ORs were not calculated when any cell values are zero; S = Schmorl’s nodes; Ap = Apophyseal facets; Ost = Vertebral body marginal osteophytosis; Bold face indicates p-values less than 0.05.
