# Supplementary material for: Osteoarthritis, labour division, and occupational specialization of the Late Shang China - insights from Yinxu (ca. 1250 - 1046 B.C.)
Source: PLoS One. 2017 May 2;12(5):e0176329. doi: 10.1371/journal.pone.0176329 (PMC5413014; doi:10.1371/journal.pone.0176329)
Supplement: S12 Table — (DOCX) [file pone.0176329.s012.docx]

**S12 Table. Odds ratio results for the comparison of osteoarthritis prevalence in females between Xin’anzhuang and Xiaomintun.**

| **Female Joint systems*** | | | **OR_20-34_** | **OR_≥ 35_** | **OR_MH_** | ***P*** | **χ^2^** | **df** | **Interpretation**  **Xin’anzhuang (AXA) vs. Xiaomintun (XMT)** |
| --- | --- | --- | --- | --- | --- | --- | --- | --- | --- |
| **Upper limb** | | **Shoulder** | — | 0.429 | 0.786 | *0.853* | 0.225 | 1 | 1.27 times XMT > AXA |
|  | | **Elbow** | — | — | — | *—* | — | — | — |
|  | | **Wrist** | — | — | — | *—* | — | — | — |
|  | | **Hand** | — | — | — | *—* | — | — | — |
| **Lower limb** | | **Hip** | — | 0.348 | 0.860 | *0.902* | 0.243 | 1 | 1.16 times XMT > AXA |
|  | | **Knee** | — | 0.333 | 0.652 | *0.563* | 0.034 | 1 | 1.53 times XMT > AXA |
|  | | **Ankle** | — | — | — | *—* | — | — | — |
|  | | **Foot** | 0.818 | 0.733 | 0.771 | *0.715* | 0.000 | 1 | 1.30 times XMT > AXA |
| **Spine** | **Cervical** | **S** | — | — | — | *—* | — | — | — |
|  |  | **Ap** | — | 0.400 | 0.246 | *0.157* | 0.991 | 1 | 4.07 times XMT > AXA |
|  |  | **Ost** | — | 2.000 | 2.000 | *0.571* | 0.001 | 1 | 2.00 times AXA > XMT |
|  | **Thoracic** | **S** | 0.188 | 0.278 | 0.236 | *0.068* | 2.155 | 1 | 4.24 times XMT > AXA |
|  |  | **Ap** | — | 0.231 | 0.231 | *0.267* | 0.266 | 1 | 4.33 times XMT > AXA |
|  |  | **Ost** | — | 0.400 | 0.525 | *0.449* | 0.094 | 1 | 1.90 times XMT > AXA |
|  | **Lumbar** | **S** | 0.643 | 0.154 | 0.292 | *0.149* | 1.021 | 1 | 3.42 times XMT > AXA |
|  |  | **Ap** | — | 0.545 | 0.349 | *0.219* | 0.735 | 1 | 2.87 times XMT > AXA |
|  |  | **Ost** | **0.400** | **0.159** | **0.209** | ***0.043*** | **2.702** | **1** | **4.78 times XMT > AXA** |

* OR_20-34,_ the odds ratio for young adults (20-34 years); OR_≥ 35,_ the odds ratio for older adults (≥ 35 years); OR_MH_, the Mantel-Haenszel common odds ratio of each joint system; — ORs were not calculated when any cell values are zero; S = Schmorl’s nodes; Ap = Apophyseal facets; Ost = Vertebral body marginal osteophytosis: Bold face indicates p-values less than 0.05.
